# ﻿Seven new species of the subgenus *Homoneura* Malloch (Diptera, Lauxaniidae, *Homoneura*) from Jiangjin District, southwestern Chongqing, China

**DOI:** 10.3897/zookeys.1206.124892

**Published:** 2024-07-02

**Authors:** Xulong Chen, Pengyan You, Wenliang Li, Zhisheng Zhang

**Affiliations:** 1 Key Laboratory of Eco-environments in Three Gorges Reservoir Region (Ministry of Education), School of Life Sciences, Southwest University, Chongqing 400715, China Southwest University Chongqing China; 2 College of Horticulture and Plant Protection, Henan University of Science and Technology, Luoyang, Henan 471023, China Henan University of Science and Technology Luoyang China

**Keywords:** Homoneurinae, illustration, morphology, new taxon, taxonomy

## Abstract

Seven new species of the subgenus Homoneura are described, Homoneura (Homoneura) biconica Chen & Li, **sp. nov.**, Homoneura (Homoneura) dilatata Chen & Li, **sp. nov.**, Homoneura (Homoneura) jiangjinensis Chen & Li, **sp. nov.**, Homoneura (Homoneura) microtricha Chen & Li, **sp. nov.**, Homoneura (Homoneura) multiseta Chen & Li, **sp. nov.**, Homoneura (Homoneura) serrulata Chen & Li, **sp. nov.**, Homoneura (Homoneura) simianshana Chen & Li, **sp. nov.**, which were collected from Jiangjin District, southwestern Chongqing, China and are assigned to the *henanensis* group. A key to all of the 53 species of this species group in China is presented.

## ﻿Introduction

The Lauxaniid fauna in southwestern China is relatively well-known, but new species are continuously discovered in the humid parts and especially in southern Yunnan and southwestern Guizhou, where significantly higher species richness and endemism are found (Gao and Yang 2002, 2005; Shi et al. 2009, 2017a, 2020; Li and Yang 2012, 2013, 2015; Shi and Yang 2015; Li et al. 2019, 2020a, 2020b, 2020c, 2020d, 2020e, 2020f, 2021a, 2021b, 2023; Zhao et al. 2022). Compared to these areas, research on the diversity of lauxaniid flies in Chongqing is relatively weak, and currently only nine species in five genera are known; four *Homoneura* species were described more recently by You et al. (2023) from Yintiaoling Nature Reserve, Chongqing, China.

Jiangjin District is located in southwestern Chongqing, adjacent to Guizhou Province to the southeast and Sichuan Province to the west and southwest. The geographical coordinates are between 105°49'–106°38' east longitude and 28°28'–29°28' north latitude. It has a subtropical humid monsoon climate, with a mild climate and abundant precipitation, high vegetation abundance and rich humus and fungi, providing a good environment for the habitat and reproduction of many organisms.

Homoneura (Homoneura) Malloch, 1927 is the largest subgenus of genus *Homoneura* Wulp, 1891 with more than 700 species distributed worldwide. The Chinese fauna of H. (Homoneura) is richly represented with more than 220 species, which are sorted into 21 species groups based on external characters and male genitalia morphology (Shi and Yang 2014). Seven new species described in this paper are assigned to the *henanensis* group of the subgenus Homoneura Malloch by the the wing having five brown spots, which are respectively located between the r-m and apical spot on R_4+5_, crossvein dm-cu and the tips of R_2+3_, R_4+5_, and M_1_.

At present, there are 46 known species of *henanensis* group in China (Kertész 1915; Matsumura 1916; Malloch 1926; Yang et al. 1999, 2001, 2003; Gao and Yang 2002, 2003, 2004, 2005; Wang et al. 2012; Papp and Gaimari 2013; Shi and Yang 2014; Shi et al. 2017b; Gao and Shi 2019; Chen and Li 2022; You et al. 2023). Seven new species were found among recently collected specimens from Jiangjin District, Chongqing in southwestern China. In present paper, the descriptions and illustrations of male genitalia of these new species are provided, increasing the number of species of the *Homoneurahenanensis* group to 53 species in China, with 11 in Chongqing.

## ﻿Materials and methods

General terminology follows Cumming and Wood (2017) and Gaimari and Miller (2021). Genitalia preparations were made by removing and macerating the apical portion of the abdomen in pancreatin for six hours (Álvarez-Padilla and Hormiga 2007), then rinsing them with distilled water for dissection and study. After examination in glycerin, they were transferred to fresh glycerin and stored in a microvial pinned below the specimen. Specimens examined were deposited in the Henan University of Science and Technology, Luoyang, Henan, China (**HAUST**).

## ﻿Taxonomy

### ﻿Key to species of Homoneura (Homoneura) henanensis group in China (modified from Gao and Shi 2019; You et al. 2023)

**Table:** 

1	Wing with brown spot at tip of Sc and R_1_ elongating along costal margin	**2**
–	Wing without brown spot at tip of Sc and R_1_	**3**
2	Basal edge of brown apical spot on R_2+3_ behind vertical level as crossvein dm-cu; wing with brown spot at tip of Sc and R_1_ slightly elongating along costal margin; surstylus claviform with 3 long setulae, postgonite long coniform with 5 short setulae	**H. (H.) hirayamae (Matsumura, 1916)**
–	Basal edge of brown apical spot on R_2+3_ at same vertical level as crossvein dm-cu; wing with brown spot at tip of Sc and R_1_, extending closely to brown apical spot on R_2+3_ along costal margin; surstylus without long setula, short claviform with a subapical concavity in posterior view; postgonite hook-like and sharp at apex	**H. (H.) similicurvata Gao & Shi, 2019**
3	Basal edge of brown apical spot on R_2+3_ at same vertical level as crossvein dm-cu	**4**
–	Basal edge of brown apical spot on R_2+3_ behind vertical level as crossvein dm-cu	**15**
4	Palpus yellow except for black at tip; surstylus broad, sheet-like with short apical setulae in lateral view and curved apically in posterior view	**H. (H.) dadongshanica Shi & Yang, 2014**
–	Palpus entirely yellow; surstylus not as above	**5**
5	Mesonotum with acrostichal setulae in 6 irregular rows	**6**
–	Mesonotum with acrostichal setulae in 8–10 irregular rows	**7**
6	Arista with longest ray slightly < 1/2 height of first flagellomere; surstylus with a small triangular process with several setulae in lateral view	**H. (H.) dagupingensis Gao & Shi, 2019**
–	Arista with longest ray as long as height of first flagellomere; surstylus with a long digitiform process in lateral view and without subapical concavity	**H. (H.) wuxica You, Chen & Li, 2023**
7	Mesonotum with acrostichal setulae in 8 irregular rows	**H. (H.) brevis Gao & Yang, 2004**
–	Mesonotum with acrostichal setulae in 10 irregular rows	**8**
8	Wing with brown apical spots on R_2+3_, R_4+5_, and M_1_ entirely separated (Fig. 22)	**H. (H.) jiangjinensis sp. nov.**
–	Wing with brown apical spots on R_2+3_, R_4+5_, and M_1_ confluent, or brown apical spots on R_4+5_ and M_1_ confluent, separated from apical spot on R_2+3_	**9**
9	Subcostal cell hyaline (Fig. 42)	**H. (H.) multiseta sp. nov.**
–	Subcostal cell pale brown	**10**
10	Ultimate and penultimate sections of M_1_ in proportion of 1.2: 1	**H. (H.) yaromi Yang, Hu & Zhu, 2001**
–	Ultimate and penultimate sections of M_1_ in proportion of 1: 1	**11**
11	Brown apical spots on R_4+5_ and M_1_ confluent, separated from apical spot on R_2+3_	**12**
–	Brown apical spots on R_2+3_, R_4+5_, and M_1_ slightly confluent and forming pale brown connecting area between 3 apical spots	**13**
12	Abdominal tergites 2–5 without brown posterior margin; syntergosternite with 2 or 3 setulae around spiracle; surstylus consisting of wide knife-like process and triangular process in lateral view; postgonites symmetrical in ventral view	**H. (H.) stepheni Shi, Gao & Shen, 2017**
–	Abdominal tergites 2–5 each with brown posterior margin; syntergosternite without setula around spiracle; surstylus long and furcated in lateral view; postgonites asymmetrical in ventral view	**H. (H.) anadaequata Gao & Shi, 2019**
13	Male tergites 2–5 each with blackish brown posterior margin (Fig. 55); ctenidium with 14 short setae on fore femur; inner process of surstylus evaginable apically with serrulate margin in posterior view (Fig. 57)	**H. (H.) serrulata sp. nov.**
–	Male tergites 2–5 each with brown posterior margin; ctenidium with 18–20 short setae on fore femur; surstylus not as above	**14**
14	Fore femur with 8 posterior dorsal setae, 6 posterior ventral setae; mid femur with 6 or 7 anterior setae; brown apical spots on R_2+3_ longer, at least 2/3 length of ultimate section of M_1_; brown median spot on R_4+5_ at middle point of distance between r-m and dm-cu	**H. (H.) shunhuangshana Chen & Li, 2022**
–	Fore femur with 9 posterior dorsal setae, 4 posterior ventral setae; mid femur with 5 anterior setae; brown apical spots on R_2+3_ shorter, as long as 1/2 length of ultimate section of M_1_ (Fig. 62); brown median spot on R_4+5_ behind middle point of distance between r-m and dm-cu (Fig. 62)	**H. (H.) simianshanica sp. nov.**
15	Basal edge of brown apical spot on R_2+3_ at same vertical level as apical spot on R_4+5_; apical spot on R_4+5_ close to brown spot on crossvein dm-cu or at least 2/3 length of ultimate section of M_1_	**16**
–	Basal edge of brown apical spot on R_4+5_ behind vertical level as apical spot on R_2+3_; apical spot on R_4+5_ far from brown spot on crossvein dm-cu and < 2/3 length of ultimate section of M_1_	**19**
16	Apical spot on R_4+5_ close to brown spot on crossvein dm-cu; ctenidium with 16 short setae on fore femur; surstylus apically acute in lateral view; pregonite absent; postgonite consisting of a furcated process and a subuliform process in ventral view	**H. (H.) denticulata Shi & Yang, 2014**
–	Apical spot on R_4+5_ ~ 2/3 length of ultimate section of M_1_, not close to brown spot on crossvein dm-cu; ctenidium with 12–14 short setae on fore femur; surstylus apically blunt in lateral view, pregonite with inverse U-shaped process and postgonite consisting a pair of subuliform processes in ventral view	**17**
17	Hypandrium with a short ventral process; pregonite with a pair of inverse U-shaped processes in ventral view; postgonite short and acute, but pregonite longer than postgonite in ventral view	**18**
–	Hypandrium with a long ventral process; shape of pregonite and postgonite not as above, but pregonite shorter than postgonite in ventral view	**H. (H.) pseudograndis Papp & Gaimari, 2013**
18	Phallus with a pair of lateral teeth subapically in ventral view; 2 arms of inverse U-shaped pregonite asymmetrical distinctly	**H. (H.) simigrandis Shi & Yang, 2014**
–	Phallus without a pair of lateral teeth subapically in ventral view; 2 arms of inverse U-shaped pregonite almost symmetrical in length	**H. (H.) grandis (Kertész, 1915)**
19	Wing with brown string-like spot on R_2+3_ and apical spots on R_4+5_ and M_1_; epandrium slender and surstylus apically acute with a long seta in lateral view	**H. (H.) curvispina Gao & Yang, 2003**
–	Wing with round, elliptical or quadrate spot on R_2+3_, R_4+5_, and M_1_; epandrium and surstylus not as above	**20**
20	Wing with brown apical spots on R_2+3_, R_4+5_, and M_1_ entirely confluent, or slightly confluent and forming pale brown connecting area between apical spots on R_2+3_, R_4+5_, and M_1_	**21**
–	Wing with brown apical spots on R_4+5_ and M_1_ confluent, separated from apical spot on R_2+3_, or apical spots on R_2+3_, R_4+5_, and M_1_ entirely separated	**27**
21	Brown medial spot on R_4+5_ separated from brown cloud on crossvein dm-cu	**22**
–	Brown medial spot on R_4+5_ confluent with brown cloud on crossvein dm-cu	**25**
22	Abdominal tergites 2–5 without blackish brown posterior margin; syntergosternite with long hairs around spiracle	**23**
–	Abdominal tergites 2–5 with blackish brown posterior margin; syntergosternite without long hair around spiracle	**24**
23	Mesonotum with acrostichal setulae in 8 irregular rows; fore femur with 3 posterior ventral setae; syntergosternite without ventral process; surstylus blunt apically; phallapodeme normal apically	**H. (H.) martini Shi, Gao & Shen, 2017**
–	Mesonotum with acrostichal setulae in 10 rows (Fig. 14); fore femur with 5 posterior ventral setae (Fig. 13); syntergosternite with a trapeziform ventral process (Fig. 18); surstylus furcated into 2 curved, short processes in lateral view (Fig. 16); phallapodeme expanded apically (Fig. 20)	**H. (H.) dilatata sp. nov.**
24	Mesonotum with acrostichal setulae in 8 irregular rows; fore femur with 10 posterior dorsal setae and ctenidium with 12 short setae; subcostal cell pale brown apically; surstylus with concavity apically in lateral view	**H. (H.) apiconcava You, Chen & Li, 2023**
–	Mesonotum with acrostichal setulae in 10 irregular rows (Fig. 4); fore femur with 8 posterior dorsal setae and ctenidium with 16 short setae (Fig. 3); subcostal cell hyaline (Fig. 2); surstylus consisting of a longer subuliform process and a shorter subuliform process in lateral view (Fig. 6)	**H. (H.) biconica sp. nov.**
25	Fore femur with 4 posteroventral setae; syntergosternite circular	**26**
–	Fore femur with 6 posteroventral setae; syntergosternite semicircular	**H. (H.) yangi Gao & Yang, 2005**
26	Abdominal tergites 2–5 without blackish brown posterior margin; surstylus indistinct, blunt apically; hypandrium Y-shaped; phallus without triangular median process in ventral view	**H. (H.) guizhouensis Gao & Yang, 2002**
–	Abdominal tergites 2–5 with blackish brown posterior margin; surstylus distinctly digitiform in lateral view; hypandrium H-shaped; phallus with a pair of triangular median process in ventral view	**H. (H.) yintiaolingica You, Chen & Li, 2023**
27	Wing with brown apical spot on R_4+5_ and M_1_ slightly confluent and forming pale brown connecting area between 2 apical spots; apical spot on R_2+3_ distinctly separated from apical spot on R_4+5_	**28**
–	Wing with brown apical spots on R_2+3_, R_4+5_, and M_1_ entirely separated	**42**
28	Mesonotum with acrostichal setulae in 10 rows	**29**
–	Mesonotum with acrostichal setulae in 6–8 rows	**32**
29	Subcostal cell hyaline	**30**
–	Subcostal cell pale brown or brown apically	**31**
30	Surstylus bulged claviform, with long setulae in lateral view; abdominal tergites 2–5 with pale brown posterior margin; arista with longest ray as long as height of first flagellomere; ctenidium with 16 or 17 short setae on fore femur; hypandrium Y-shaped	**H. (H.) bispinalis Yang, Hu & Zhu, 2001**
–	Surstylus T-shaped and rounded apically in lateral view; abdominal tergites 2–5 without pale brown posterior margin; arista with longest ray shorter than height of first flagellomere; ctenidium with 10 short setae on fore femur; hypandrium H-shaped	**H. (H.) fujianensis Yang, Zhu & Hu, 2003**
31	Mesoscutum with 1 square or oval brown spot before scutoscutellar suture, scutellum with 1 square brown spot at middle; fore femur with 7 or 8 posterior dorsal setae, ctenidium with 22 short setae; surstylus long and spine-like in lateral view, without inner process	**H. (H.) maculiscutellata Chen & Li, 2022**
–	Mesoscutum without spot before scutoscutellar suture, scutellum without brown spot; fore femur with 5 posterior dorsal setae, ctenidium with 12 short setae; surstylus with 1 short, claviform inner process in lateral view	**H. (H.) tianeensis Gao & Yang, 2004**
32	Abdomen yellow or pale brown, at least tergites 2–5 with black or brown posterior margin	**33**
–	Abdomen yellow, tergites 1–6 without brown posterior margin	**35**
33	Abdomen pale brown; surstylus straight and claviform in lateral view	**H. (H.) serrata Gao & Yang, 2002**
–	Abdomen yellow; surstylus subuliform in lateral view	**34**
34	Arista with longest ray as long as 1/2 height of first flagellomere; ultimate section of CuA_1_ ~ 1/9 of penultimate; hypandrium inverse U-shaped; postgonite short, with 2 teeth-like processes in lateral view; phallus curved backwards apically and acute at apex in lateral view	**H. (H.) longiacutata Gao & Shi, 2019**
–	Arista with longest ray as long as height of first flagellomere (Fig. 31); ultimate section of CuA_1_ ~ 1/5 of penultimate (Fig. 32); hypandrium H-shaped (Fig. 39); postgonite long spine-like in lateral view (Fig. 40); phallus not curved backwards apically, without acute tip in lateral view (Fig. 40)	**H. (H.) microtricha sp. nov.**
35	Mid femur with 5 or 6 anterior setae	**36**
–	Mid femur with 4 anterior setae	**39**
36	Mesonotum with acrostichal setulae in 6 rows	**37**
–	Mesonotum with acrostichal setulae in 8 rows	**38**
37	Arista with longest ray as long as 4/5 height of first flagellomere; surstylus long and curved at apex in lateral view	**H. (H.) longicurva Gao & Shi, 2019**
–	Arista with longest ray slightly shorter than height of first flagellomere; surstylus short and narrow in lateral view	**H. (H.) chongqingensis You, Chen & Li, 2023**
38	Fore femur with 6 posterior dorsal setae, 2 posterior ventral setae and ctenidium with 12 short setae; surstylus consisting of a small acute apical process, directed downward and a slender knife-like process with dense setulae on dorsal margin in lateral view	**H. (H.) henanensis Yang, Zhu & Hu, 1999**
–	Fore femur with 8 posterior dorsal setae, 4 posterior ventral setae and ctenidium with 15–17 short setae; surstylus claviform in lateral view	**H. (H.) pangae Shi, Gao & Shen, 2017**
39	Wing with a brown spot between r-m and apical spot on R_4+5_ distinctly or slightly confluent with brown spot on crossvein dm-cu; surstylus claviform or digitiform	**40**
–	Wing with a brown quadrate spot between r-m and apical spot on R_4+5_ separated from brown spot on crossvein dm-cu; surstylus not as above	**41**
40	Ctenidium with 15 short setae on fore femur; surstylus absent; pregonite short, broad, and acute apically in ventral view; postgonite consisting of a furcated process and a subuliform process in ventral view	**H. (H.) curvispinosa Yang, Hu & Zhu, 2001**
–	Ctenidium with 13 short setae on fore femur; surstylus digitiform with long setulae in lateral view; pregonite and postgonite furcated apically, pregonite shorter than postgonite in ventral view	**H. (H.) zonalis Yang, Zhu & Hu, 1999**
41	Fore femur with 3 posteroventral setae; epandrium blunt triangular apically; surstylus separated from epandrium and originated from anterior ventral corner of epandrium, with dense tiny setulae on apical 2/3	**H. (H.) tianjingshanica Shi & Yang, 2014**
–	Fore femur with 4 posteroventral setae; epandrium and surstylus fused, blunt round apically	**H. (H.) tianmushana Yang, Hu & Zhu, 2001**
42	Ctenidium with 17–19 short setae on fore femur	**43**
–	Ctenidium with 10–16 short setae on fore femur	**44**
43	The first flagellomere ~ 1.8 × longer than high; surstylus narrow and columnar in lateral view and broad with tiny setulae in posterior view; postgonites triangular with sharp apex in lateral view	**H. (H.) zhangjiajiensis Shi & Yang, 2014**
–	The first flagellomere ~ 2.3 × longer than high; surstylus light color and narrow at base while dark yellow and broad at apex, nearly trapeziform with 2 long setulae in lateral view; postgonite hook-like in lateral view	**H. (H.) bicolorata Gao & Shi, 2019**
44	Pregonite and postgonite subuliform in ventral view or short and triangular in lateral view	**45**
–	Pregonite and postgonite not as above	**47**
45	Surstylus without acute or triangular process, blunt and rounded apically and slightly rolled up with several setulae in lateral view	**H. (H.) miaoae Gao & Shi, 2019**
–	Surstylus with acute or triangular process, not as above in lateral view	**46**
46	Surstylus very broad ball-like with a triangular process apically in lateral view; hypandrium H-shaped; phallus acute subapically in lateral view	**H. (H.) kuankuoshuiensis Wang & Yang, 2012**
–	Surstylus narrow, acute apically in lateral view; hypandrium Y-shaped; phallus blunt and subapically rounded in lateral view	**H. (H.) chinensis Malloch, 1926**
47	Subcostal cell hyaline or pale yellow apically	**48**
–	Subcostal cell dark apically	**50**
48	Mesonotum with acrostichal setulae in 6 regular rows; surstylus curved and knife-like in lateral view; postgonite absent	**H. (H.) spectabilis Gao & Shi, 2019**
–	Mesonotum with acrostichal setulae in 10 irregular rows; surstylus not as above	**49**
49	Basal edge of brown apical spot on R_4+5_ at same vertical level as apical spot on M_1_; surstylus consisting of a slender knife-shaped process and a furcated process with several setulae on subapical and apical margin and a small tooth on lateral margin in lateral view	**H. (H.) caoi Wang & Yang, 2012**
–	Basal edge of brown apical spot on R_4+5_ behind vertical level as apical spot on M_1_; surstylus short and broad, with a row of long apical setulae	**H. (H.) jiangjinensis Shi, Gao & Shen, 2017**
50	Syntergosternite elliptic without sternal part flat; surstylus broad and slightly curved apically in lateral view	**H. (H.) platimarginata Gao & Shi, 2019**
–	Syntergosternite circular, but with sternal part flat; surstylus not as above	**51**
51	Mesonotum with acrostichal setulae in 8 rows	**H. (H.) curvata Yang, Zhu & Hu, 1999**
–	Mesonotum with acrostichal setulae in 10 irregular rows	**52**
52	A brown elliptical spot present between r-m and apical spot on R_4+5_; mid femur with 6–8 anterior setae; surstylus curved knife-like, acute apically in lateral view; postgonite longer than phallus, elongate subuliform, curved forwards apically in lateral view	**H. (H.) longispina Gao & Yang, 2004**
–	A brown square spot present between r-m and apical spot on R_4+5_; mid femur with 4 anterior setae; surstylus short, triangular, and acute apically, with several long setae on dorsal margin and a row of short setulae on ventral margin in lateral view; both pregonite and postgonite shorter subuliform, ~ ½ length of phallus in ventral view	**H. (H.) acutata Yang, Zhu & Hu, 1999**

### ﻿Species descriptions

#### ﻿ Homoneura (Homoneura) biconica

Taxon classificationAnimaliaDipteraLauxaniidae

Chen & Li sp. nov.

34DDBAE2-60AA-5998-B41B-DAA5F5E28D1E

https://zoobank.org/FA24E555-3C34-4BC7-8DBA-8C8BCA604CC2

Figs 1–5 , 6–10 Chinese name: 双锥同脉缟蝇

##### Type material.

***Holotype***: ♂, **China**, Chongqing City, Jiangjin District, Simianshan Natural Reserve, Zhengqiangou, 28°36'59.54"N, 106°26'25.88"E, 1273 m, 14.VI.2022, leg. Xulong Chen. ***Paratypes***: 6♂♂, same data as holotype; 1♂, **China**, Chongqing City, Jiangjin District, Simianshan Natural Reserve, Dahonghai, 28°35'34.27"N, 106°26'34.93"E, 1144 m, 15.VII.2022, leg. Xulong Chen.

##### Etymology.

The specific name comes from the combination of the prefix *bi*- (meaning two) and the Latin word, *conica* (meaning cone-shaped), referring to the surstylus consisting of two subuliform processes in lateral view.

##### Diagnosis.

Mesonotum with acrostichal setulae in ten irregular rows. Basal margin of brown apical spot on R_2+3_ behind vertical level as crossvein dm-cu; brown apical spots on R_2+3_, R_4+5_, and M_1_ slightly confluent; subcostal cell hyaline. Male tergites 2–5 with blackish brown posterior margin. Surstylus consisting of two subuliform processes in lateral view. Pregonite and postgonite inwardly curved, pregonite with one long setula. Phallus long and knife-like with an acute subapical tooth in lateral view.

##### Description.

**Male.** Body length 8.4–8.6 mm, wing length 8.3–8.4 mm.

**Figures 1–5.**
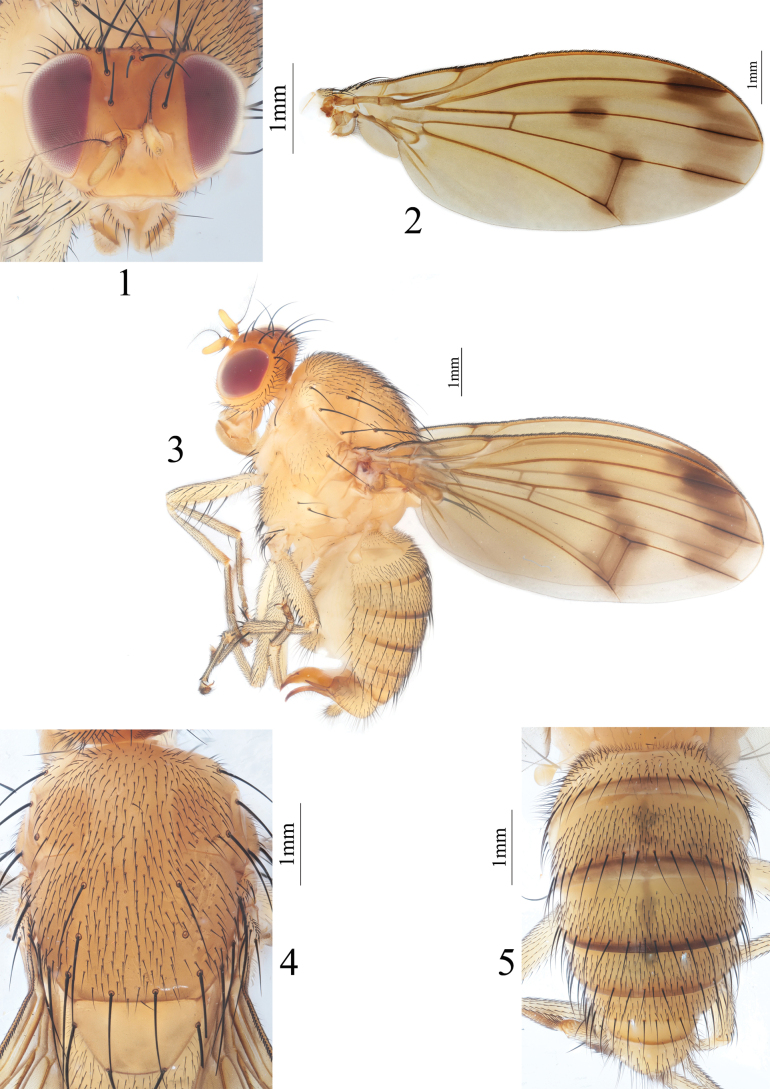
Homoneura (Homoneura) biconica sp. nov. male **1** head, anterior view **2** wing **3** habitus, lateral view **4** thorax, dorsal view **5** abdomen, dorsal view.

***Head*** (Fig. 1) yellow. Frons as long as wide and parallel-sided; ocellar triangle yellow, ocellar seta developed, longer than anterior fronto-orbital seta, anterior fronto-orbital seta shorter than posterior fronto-orbital seta. Gena ~ 1/7 height of eye. Antenna yellow, first flagellomere ~ 2.0 × longer than high; arista black except pale brown at base, long plumose, with longest ray as long as height of first flagellomere. Proboscis and palpus yellow.

***Thorax*** (Fig. 4) yellow, with gray pruinosity. 0+3 dorsocentral setae, anteriormost postsutural dorsocentral seta far from scutal suture, acrostichal setulae in ten irregular rows. Legs yellow. Fore femur with eight posterior dorsal setae, five posterior ventral setae and ctenidium with 16 short setae; fore tibia with one dorsal preapical seta and one short apical ventral seta. Mid femur with five or six anterior setae and one apical posterior seta; mid tibia with one dorsal preapical seta and three strong apical ventral setae. Hind femur with several weak anterior ventral setae and one preapical anterior dorsal seta; hind tibia with one weak dorsal preapical seta and one short apical ventral seta. Wing (Fig. 2) slightly yellow, basal margin of brown apical spot on R_2+3_ behind vertical level as crossvein dm-cu; brown apical spots on R_2+3_, R_4+5_, and M_1_ slightly confluent and forming pale brown connecting area between apical spots on R_2+3_, R_4+5_, and M_1_; brown median spot on R_4+5_ separated from brown cloud-like spot on crossvein dm-cu; subcostal cell hyaline; eight short hairs present at base of R_4+5_; costa with 2^nd^ (between R_1_ and R_2+3_), 3^rd^ (between R_2+3_ and R_4+5_), and 4^th^ (between R_4+5_ and M_1_) sections in proportion of 4: 1: 0.8; r-m before middle of discal cell; ultimate and penultimate sections of M_1_ in proportion of 3.5: 3.3; ultimate section of CuA_1_ ~ 1/8 of penultimate. Haltere yellow.

**Figures 6–10.**
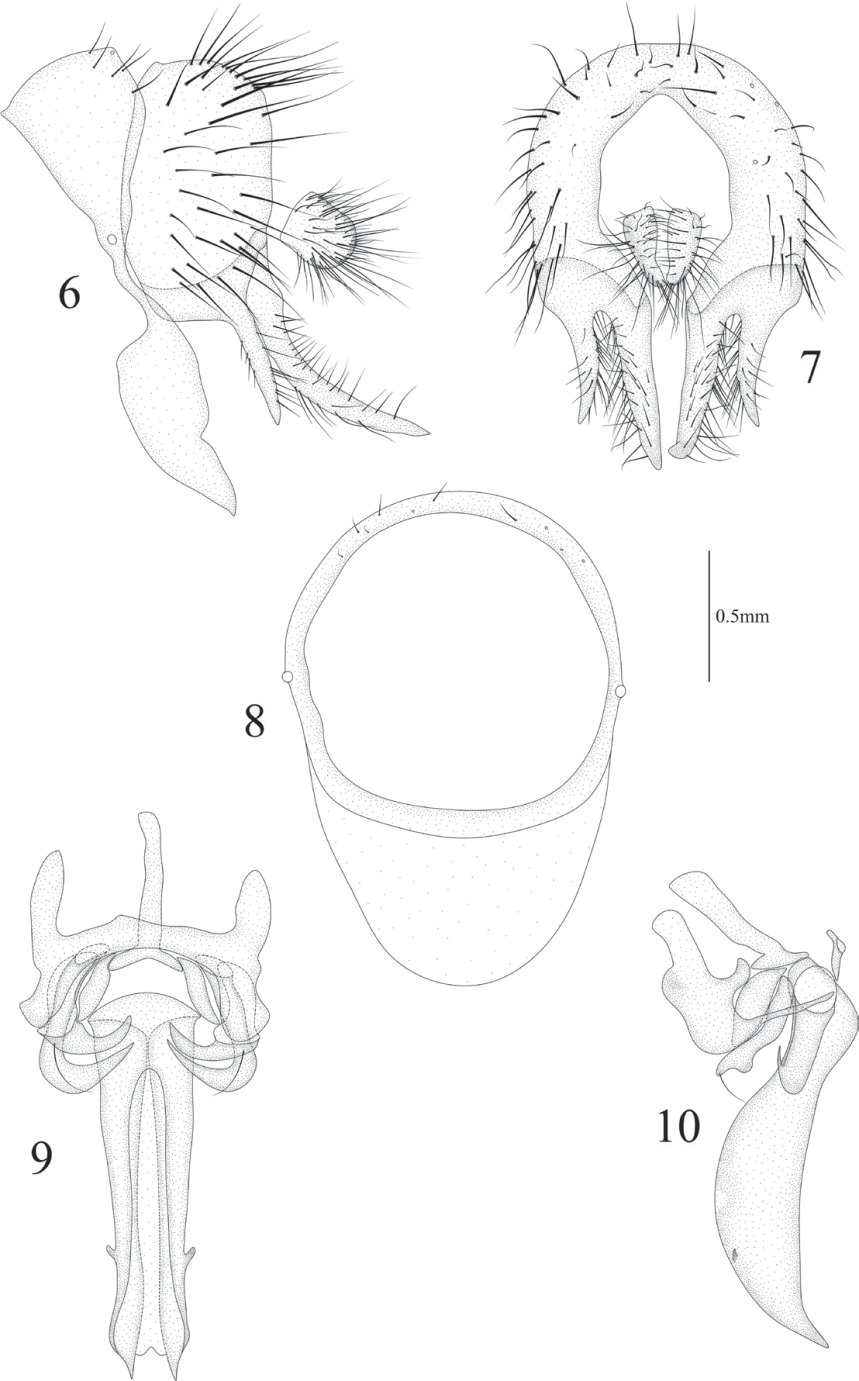
Homoneura (Homoneura) biconica sp. nov. male **6** syntergosternite and epandrium, lateral view 7 epandrial complex, posterior view **8** syntergosternite, anterior view **9** phallic complex, ventral view **10** phallic complex, lateral view. Scale bar: 0.5 mm.

***Abdomen*** (Fig. 5) yellow, tergites 2–5 with blackish brown posterior margin. Male genitalia (Figs 6–10): syntergosternite circular, with a triangular ventral process and with several dorsal setulae. Epandrium broad in lateral view; surstylus consisting of a longer subuliform process and a shorter subuliform process in lateral view. Hypandrium H-shaped. Pregonite and postgonite curved inwards, pregonite with one long setula. Phallus long, knife-like, and with an acute subapical tooth in lateral view. Phallapodeme shorter than phallus.

**Female.** Unknown.

##### Distribution.

China (Chongqing).

##### Remarks.

The new species resembles Homoneura (Homoneura) apiconcava in the habitus and abdominal tergites 2–5 with blackish brown posterior margin [see You et al. 2023: figs 3, 5], but it can be distinguished from the latter by the following: mesonotum with acrostichal setulae in ten irregular rows; fore femur with eight posterior dorsal setae and ctenidium with 16 short setae; subcostal cell hyaline; surstylus consisting of a longer subuliform process and a shorter subuliform process in lateral view. In H. (H.) apiconcava, mesonotum with acrostichal setulae in eight irregular rows; fore femur with ten posterior dorsal setae and ctenidium with 12 short setae; subcostal cell pale brown apically; surstylus with concavity apically in lateral view [see You et al. 2023: figs 2–6].

#### ﻿ Homoneura (Homoneura) dilatata

Taxon classificationAnimaliaDipteraLauxaniidae

Chen & Li sp. nov.

1C2CEA6C-B3C9-5EAC-8318-081463A94673

https://zoobank.org/8E80C793-E526-48DD-B79B-88B1DF3D89BF

Figs 11–15 , 16–20 Chinese name: 膨突同脉缟蝇

##### Type material.

***Holotype***: ♂, **China**, Chongqing City, Jiangjin District, Simianshan Natural Reserve, Zhengqiangou, 28°36'59.54"N, 106°26'25.88"E, 1273 m, 14.VI.2022, leg. Xulong Chen. ***Paratypes***: 1♂, **China**, Chongqing City, Jiangjin District, Simianshan Natural Reserve, Dawopu, 28°34'11.28"N, 106°20'26.96"E, 1007 m, 6.IX.2022, leg. Xulong Chen.

##### Etymology.

The specific name refers to the phallapodeme expanded apically in lateral view.

##### Diagnosis.

Mesonotum with acrostichal setulae in ten irregular rows. Basal margin of brown apical spot on R_2+3_ behind vertical level as crossvein dm-cu; brown apical spots on R_2+3_, R_4+5_, and M_1_ slightly confluent. Syntergosternite with a setula around spiracle. Surstylus furcated into two short, curved processes in lateral view. Hypandrium with one short subuliform ventral process. Phallapodeme expanded apically in lateral view.

##### Description.

**Male.** Body length 7.7–8.1 mm, wing length 7.9–8.0 mm.

**Figures 11–15.**
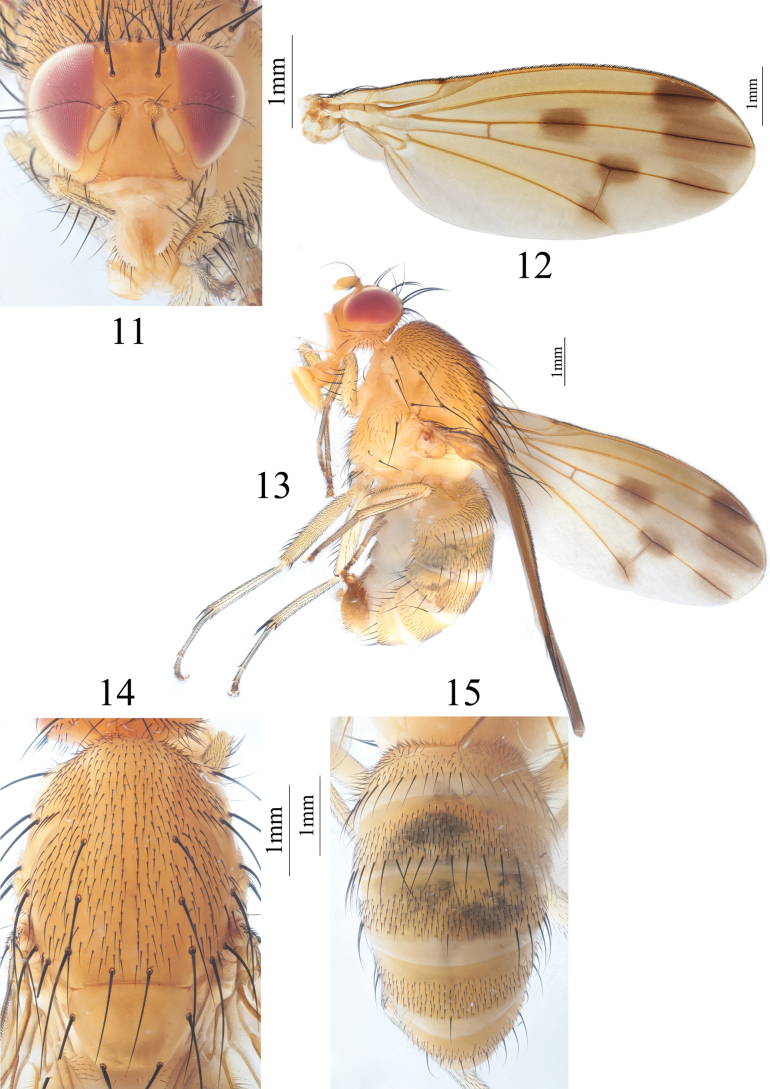
Homoneura (Homoneura) dilatata sp. nov. male **11** head, anterior view **12** wing **13** habitus, lateral view **14** thorax, dorsal view **15** abdomen, dorsal view.

***Head*** (Fig. 11) yellow. Frons as long as wide and parallel-sided; ocellar triangle yellow, ocellar seta developed, as long as anterior fronto-orbital seta, anterior fronto-orbital seta shorter than posterior fronto-orbital seta. Gena ~ 1/8 height of eye. Antenna yellow, first flagellomere ~ 2.0 × longer than high; arista black except pale brown at base, long plumose, with longest ray as long as height of first flagellomere. Proboscis and palpus yellow.

***Thorax*** (Fig. 14) yellow, with gray pruinosity. 0+3 dorsocentral setae, anteriormost postsutural dorsocentral seta far from scutal suture, acrostichal setulae in ten irregular rows. Legs pale yellow. Fore femur with nine posterior dorsal setae, five posterior ventral setae and ctenidium with 16 short setae; fore tibia with one dorsal preapical seta and one short apical ventral seta. Mid femur with five anterior setae and one apical posterior seta; mid tibia with one dorsal preapical seta and three strong apical ventral setae. Hind femur with several weak anterior ventral setae and one preapical anterior dorsal seta; hind tibia with one weak dorsal preapical seta and one short apical ventral seta. Wing (Fig. 12) slightly yellow, basal margin of brown apical spot on R_2+3_ behind vertical level as crossvein dm-cu; brown apical spots on R_2+3_, R_4+5_, and M_1_ slightly confluent and forming pale brown connecting area between apical spots on R_2+3_, R_4+5_, and M_1_; brown median spot on R_4+5_ separated from brown cloud-like spot on crossvein dm-cu; subcostal cell pale brown apically; three short hairs present at base of R_4+5_; costa with 2^nd^ (between R_1_ and R_2+3_), 3^rd^ (between R_2+3_ and R_4+5_), and 4^th^ (between R_4+5_ and M_1_) sections in proportion of 10.5: 2.7: 2.2; r-m before middle of discal cell; ultimate and penultimate sections of M_1_ in proportion of 1: 1; ultimate section of CuA_1_ ~ 1/6 of penultimate. Haltere yellow.

**Figures 16–20.**
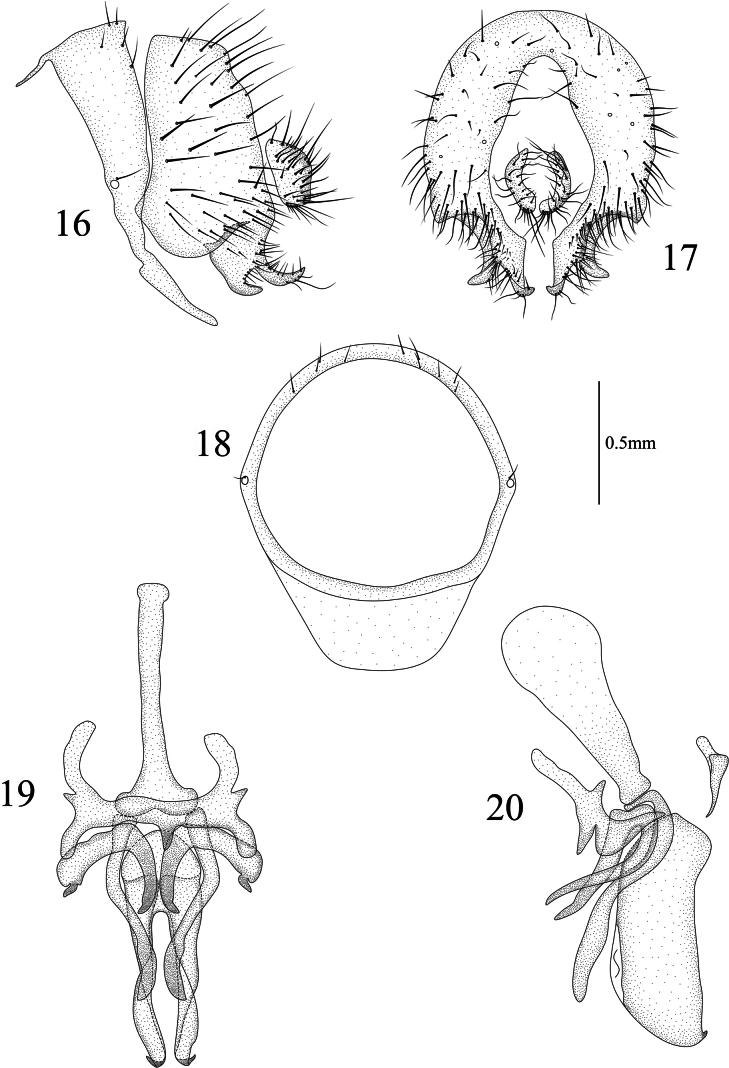
Homoneura (Homoneura) dilatata sp. nov. male **16** syntergosternite and epandrium, lateral view **17** epandrial complex, posterior view **18** syntergosternite, anterior view **19** phallic complex, ventral view **20** phallic complex, lateral view. Scale bar: 0.5 mm.

***Abdomen*** (Fig. 15) yellow. Male genitalia (Figs 16–20): syntergosternite circular with a trapeziform ventral process, with several dorsal setulae and a setula around spiracle. Epandrium broad in lateral view; surstylus hairy, furcated into two short, curved processes in lateral view. Hypandrium H-shaped, with one short subuliform ventral process. Pregonite V-shaped in ventral view, acute apically, postgonite curved, claviform. Phallus with one small, curved hook in lateral view. Phallapodeme expanded apically in lateral view, slightly shorter than phallus.

**Female.** Unknown.

##### Distribution.

China (Chongqing).

##### Remarks.

The new species resembles Homoneura (Homoneura) martini in brown apical spots on R_2+3_, R_4+5_, and M_1_ slightly confluent, abdominal tergites 2–5 without blackish brown posterior margin and syntergosternite with long hairs around spiracle [see Shi et al. 2017b: figs 10, 13, 14, 16], but it can be distinguished from the latter by the following: mesonotum with acrostichal setulae in ten rows; fore femur with five posterior ventral setae; syntergosternite with a trapeziform ventral process; surstylus furcated into two short, curved processes in lateral view; phallapodeme expanded apically. In H. (H.) martini, mesonotum with acrostichal setulae in eight irregular rows; fore femur with three posterior ventral setae; syntergosternite without ventral process; surstylus blunt apically; phallapodeme normal apically [see Shi et al. 2017b: figs 12, 16, 17, 19].

#### ﻿ Homoneura (Homoneura) jiangjinensis

Taxon classificationAnimaliaDipteraLauxaniidae

Chen & Li sp. nov.

042E9CAA-6B98-5C5D-A99D-93A124A7474D

https://zoobank.org/5EEEC111-1B29-44FD-89EC-8F7616B7D0D1

Figs 21–25 , 26–30 Chinese name: 江津同脉缟蝇

##### Type material.

***Holotype***: ♂, **China**, Chongqing City, Jiangjin District, Simianshan Natural Reserve, Zhengqiangou, 28°36'59.54"N, 106°26'25.88"E, 1273 m, 14.VI.2022, leg. Xulong Chen. ***Paratypes***: 1♂, same data as holotype.

##### Etymology.

The specific name refers to the type locality Jiangjin District.

##### Diagnosis.

Basal margin of brown apical spot on R_2+3_ at same vertical level as crossvein dm-cu; brown apical spots on R_2+3_, R_4+5_, and M_1_ entirely separated. Syntergosternite with several setulae above right spiracle, ventral process with several hairs at middle. Surstylus consisting of a long spinous process and a hairy blunt process in lateral view. Hypandrium with a pair of tooth-like ventral processes at middle. Pregonite consisting of a short spinous process and a long curved spinous process, postgonite curved and spine-like in lateral view. Phallus with a subapical tooth in lateral view.

##### Description.

**Male.** Body length 7.0–7.1 mm, wing length 6.9 mm.

**Figures 21–25.**
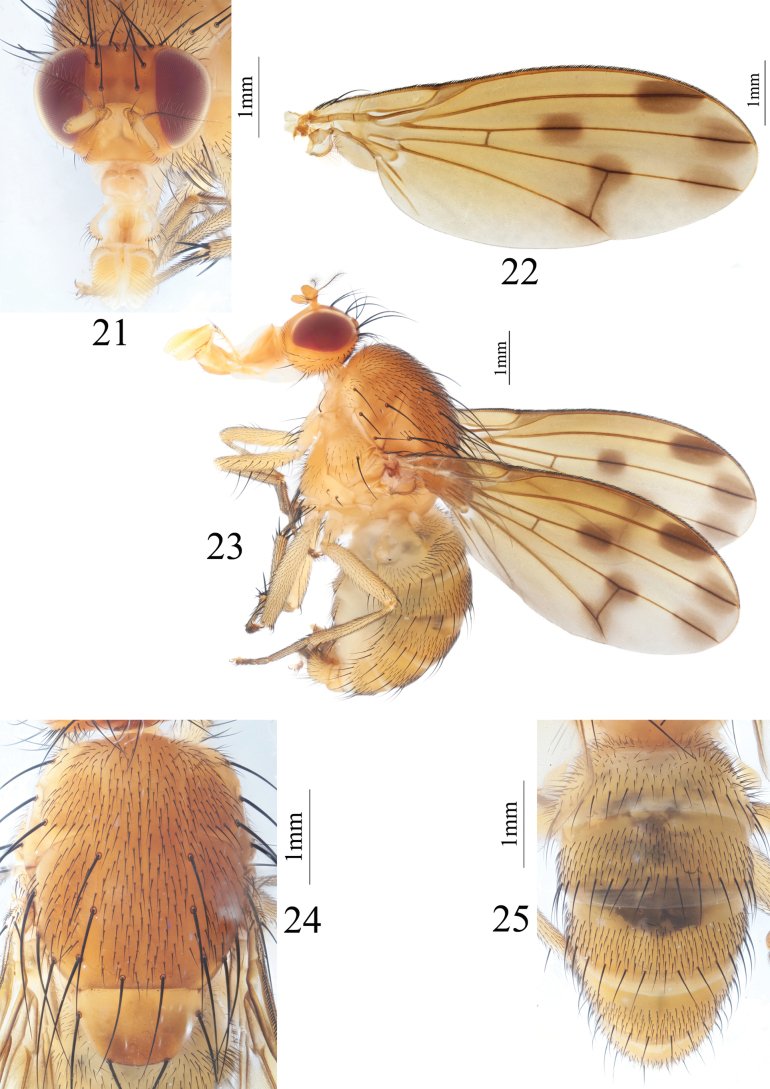
Homoneura (Homoneura) jiangjinensis sp. nov. male **21** head, anterior view **22** wing **23** habitus, lateral view **24** thorax, dorsal view **25** abdomen, dorsal view.

***Head*** (Fig. 21) yellow. Frons as long as wide and parallel-sided; ocellar triangle yellow, ocellar seta developed, longer than anterior fronto-orbital seta, anterior fronto-orbital seta shorter than posterior fronto-orbital seta. Gena ~ 1/8 height of eye. Antenna yellow, first flagellomere ~ 2.0 × longer than high; arista black except yellow at base, long plumose, with longest ray as long as height of first flagellomere. Proboscis and palpus yellow.

***Thorax*** (Fig. 24) yellow, with gray pruinosity. 0+3 dorsocentral setae, anteriormost postsutural dorsocentral seta far from scutal suture, acrostichal setulae in ten irregular rows. Legs yellow. Fore femur with ten posterior dorsal setae, five or six posterior ventral setae and ctenidium with 16 short setae; fore tibia with one dorsal preapical seta and one short apical ventral seta. Mid femur with five anterior setae and one apical posterior seta; mid tibia with one dorsal preapical seta and three strong apical ventral setae. Hind femur with several weak anterior ventral setae and one preapical anterior dorsal seta; hind tibia with one weak dorsal preapical seta and one short apical ventral seta. Wing (Fig. 22) slightly yellow, basal margin of brown apical spot on R_2+3_ at same vertical level as crossvein dm-cu; brown apical spots on R_2+3_, R_4+5_, and M_1_ entirely separated; brown median spot on R_4+5_ separated from brown cloud-like spot on crossvein dm-cu; subcostal cell pale brown apically; costa with 2^nd^ (between R_1_ and R_2+3_), 3^rd^ (between R_2+3_ and R_4+5_), and 4^th^ (between R_4+5_ and M_1_) sections in proportion of 4: 1.2: 0.8; r-m before middle of discal cell; ultimate and penultimate sections of M_1_ in proportion of 1.1: 1; ultimate section of CuA_1_ ~ 1/7 of penultimate. Haltere yellow.

**Figures 26–30.**
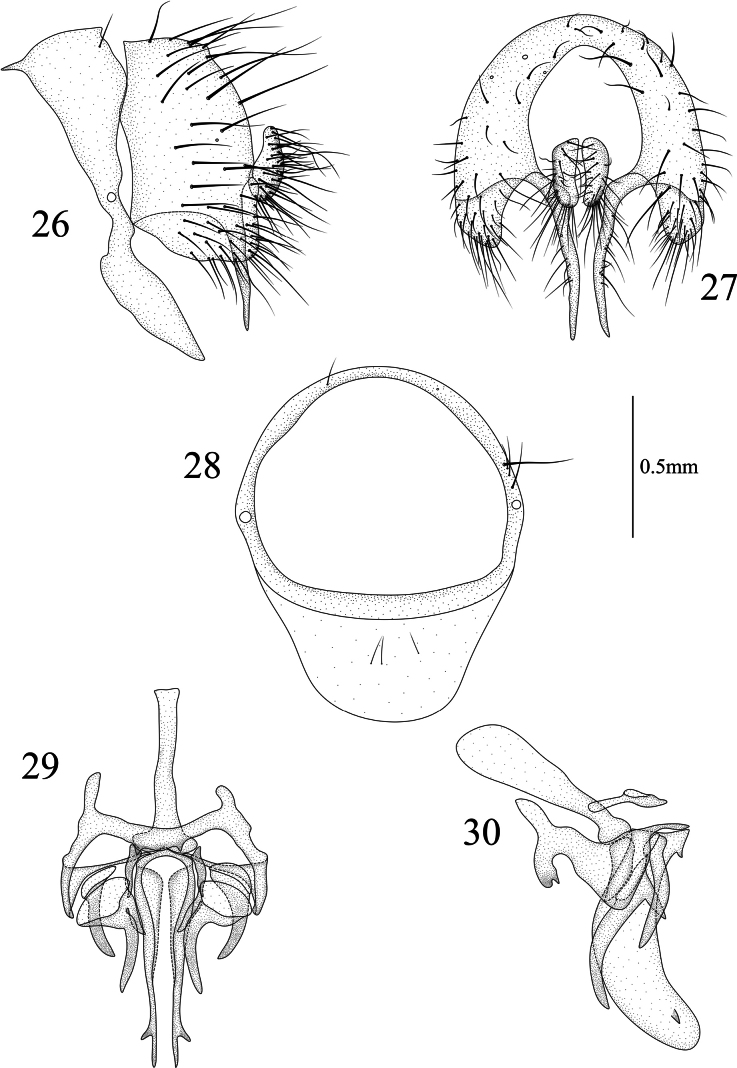
Homoneura (Homoneura) jiangjinensis sp. nov. male **26** syntergosternite and epandrium, lateral view **27** epandrial complex, posterior view **28** syntergosternite, anterior view **29** phallic complex, ventral view **30** phallic complex, lateral view. Scale bar: 0.5 mm.

***Abdomen*** (Fig. 25) yellow. Male genitalia (Figs 26–30): syntergosternite circular with a trapeziform ventral process, with several dorsal setulae and several setulae above right spiracle, ventral process with several hairs at middle. Epandrium broad in lateral view; surstylus consisting of a long spinous process and a blunt and hairy process in lateral view. Hypandrium H-shaped, with a pair of tooth-like ventral processes at middle. Pregonite consisting of a short spinous process and a long curved spinous process, postgonite curved and spine-like in lateral view. Phallus curved backwards, with a subapical tooth in lateral view. Phallapodeme shorter than phallus.

**Female.** Unknown.

##### Distribution.

China (Chongqing).

##### Remarks.

The new species resembles Homoneura (Homoneura) caoi in the habitus, mesonotum with acrostichal setulae in ten irregular rows and brown apical spots on R_2+3_, R_4+5_, and M_1_ entirely separated [see Wang et al. 2012: figs 12, 14, 15], but it can be distinguished from the latter by the following: fore femur with ten posterior dorsal setae and ctenidium with 16 short setae; subcostal cell pale brown apically; abdominal tergites 2–5 without brown posterior margin; syntergosternite with several setulae above right spiracle; surstylus consisting of a long spinous process and a blunt hairy process in lateral view; hypandrium with a pair of tooth-like ventral processes at middle; pregonite consisting of a short spinous process and a long curved spinous process, postgonite curved and spine-like in lateral view. In H. (H.) caoi, fore femur with seven or eight posterior dorsal setae and ctenidium with 12 or 13 short setae; subcostal cell hyaline; abdominal tergites 2–5 with brown posterior margin; syntergosternite without setula around spiracle; surstylus trifurcated; gonopod with three inwardly curved arc-shaped processes [see Wang et al. 2012: figs 12, 15, 16, 19, 20, 21].

#### ﻿ Homoneura (Homoneura) microtricha

Taxon classificationAnimaliaDipteraLauxaniidae

Chen & Li sp. nov.

4C5ED6FF-7976-5993-92EF-9B637FE70D26

https://zoobank.org/3070733C-4EF9-48F9-BBC2-45424FC52630

Figs 31–35 , 36–40 Chinese name: 微毛同脉缟蝇

##### Type material.

***Holotype***: ♂, **China**, Chongqing City, Jiangjin District, Simianshan Natural Reserve, Zhengqiangou, 28°36'59.54"N, 106°26'25.88"E, 1273 m, 15.VI.2022, leg. Xulong Chen. ***Paratypes***: 43♂♂6♀♀, **China**, Chongqing City, Jiangjin District, Simianshan Natural Reserve, Zhengqiangou, 28°36'59.54"N, 106°26'25.88"E, 1273 m, 14.VI.2022, leg. Xulong Chen; 1♂, **China**, Chongqing City, Jiangjin District, Simianshan Natural Reserve, Zhengqiangou, 28°36'59.54"N, 106°26'25.88"E, 1273 m, 26.VI.2022, leg. Pengyan You; 3♂♂8♀♀, **China**, Chongqing City, Jiangjin District, Simianshan Natural Reserve, Dahonghai, 28°35'34.27"N, 106°26'34.93"E, 1144 m, 15.VII.2022, leg. Xulong Chen; 5♂♂, **China**, Chongqing City, Jiangjin District, Simianshan Natural Reserve, Mohuayan, 28°35'15.67"N, 106°22'7.36"E, 1017 m, 14.VII.2022, leg. Xulong Chen; 2♂♂1♀, **China**, Chongqing City, Jiangjin District, Simianshan Natural Reserve, Zhenzhutan, 28°35'50.74"N, 106°25'25.70"E, 1226 m, 15.VII.2022, leg. Xulong Chen; 2♂♂2♀♀, **China**, Chongqing City, Jiangjin District, Simianshan Natural Reserve, Tudiyan, 28°37'24.45"N, 106°24'6.69"E, 1126 m, 15.VII.2022, leg. Xulong Chen; 1♂2♀♀, **China**, Chongqing City, Jiangjin District, Simianshan Natural Reserve, Chaoyuanguan, 28°38'53.38"N, 106°20'23.84"E, 920 m, 14. VII.2022, leg. Xulong Chen; 1♂, **China**, Chongqing City, Jiangjin District, Taihe Management and Protection Station, Heishenmiao, 28°48'9.21"N, 106°15'46.04"E, 836 m, 14.VII.2022, leg. Xulong Chen; 1♂, **China**, Chongqing City, Jiangjin District, Dayuandong National Forest Park, Kongzimiao, 28°53'9.68"N, 106°15'24.28"E, 709m, 12.VII.2022, leg. Xulong Chen; 1♂1♀, **China**, Chongqing City, Jiangjin District, Dayuandong National Forest Park, Shuijingwan, 28°53'10.96"N, 106°14'19.32"E, 717 m, 13.VII.2022, leg. Xulong Chen; 2♂♂1♀, **China**, Chongqing City, Jiangjin District, Dayuandong National Forest Park, Tian’ehu, 28°52'54.45"N, 106°15'14.53"E, 728 m, 13.VII.2022, leg. Xulong Chen; 4♂♂12♀♀, **China**, Chongqing City, Jiangjin District, Dayuandong National Forest Park, Diaojiaolou, 28°53'5.89"N, 106°15'42.18"E, 731 m, 13.VII.2022, leg. Xulong Chen.

##### Etymology.

The specific name comes from the combination of the prefix *micro*- (meaning small) and the Latin word, *tricha* (meaning hair), referring to the surstylus covered by small hairs.

##### Diagnosis.

Mesonotum with acrostichal setulae in eight irregular rows. Brown apical spots on R_4+5_ and M_1_ slightly confluent, separated from apical spot on R_2+3_; brown median spot on R_4+5_ slightly fused with brown cloud-like spot on crossvein dm-cu. Surstylus subuliform in lateral view. Pregonite knife-like in ventral view, connected with postgonite; postgonite long and spine-like in lateral view. Phallus with two pairs of subapical teeth in lateral view.

##### Description.

**Male.** Body length 5.8–6.3 mm, wing length 5.6–6.1 mm.

**Figures 31–35.**
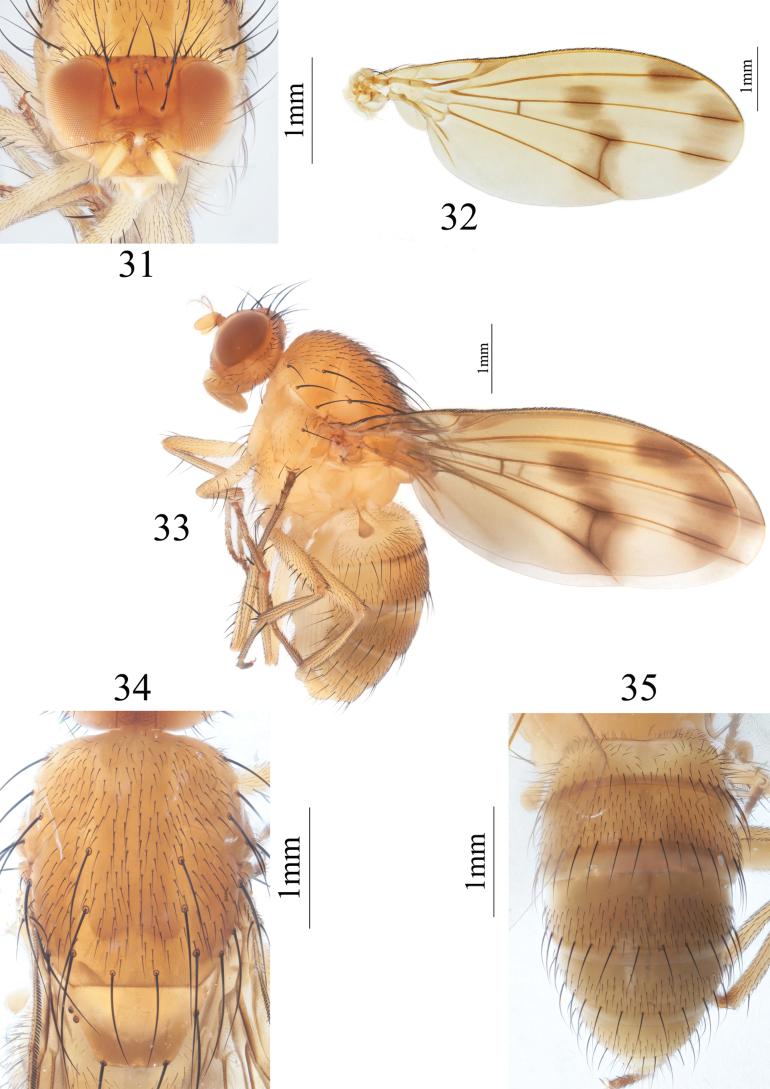
Homoneura (Homoneura) microtricha sp. nov. male **31** head, anterior view **32** wing **33** habitus, lateral view **34** thorax, dorsal view **35** abdomen, dorsal view.

***Head*** (Fig. 31) yellow. Frons as long as wide and parallel-sided; ocellar triangle yellow, ocellar seta developed, longer than anterior fronto-orbital seta, anterior fronto-orbital seta shorter than posterior fronto-orbital seta. Gena ~ 1/8 height of eye. Antenna yellow, first flagellomere ~ 2.0 × longer than high; arista black except yellow at base, long plumose, with longest ray slightly shorter than height of first flagellomere. Proboscis pale yellow, palpus yellow.

***Thorax*** (Fig. 34) yellow, with gray pruinosity. 0+3 dorsocentral setae, anteriormost postsutural dorsocentral seta far from scutal suture, acrostichal setulae in eight irregular rows. Legs yellow. Fore femur with seven posterior dorsal setae, four posterior ventral setae, and ctenidium with 14 or 15 short setae; fore tibia with one dorsal preapical seta and one short apical ventral seta. Mid femur with four or five anterior setae and one apical posterior seta; mid tibia with one dorsal preapical seta and three strong apical ventral setae. Hind femur with one preapical anterior dorsal seta; hind tibia with one weak dorsal preapical seta and one short apical ventral seta. Wing (Fig. 32) slightly yellow, basal margin of brown apical spot on R_2+3_ behind vertical level as crossvein dm-cu; brown apical spots on R_4+5_ and M_1_ slightly confluent, separated from apical spot on R_2+3_; brown median spot on R_4+5_ slightly fused with brown cloud-like spot on crossvein dm-cu; subcostal cell pale brown apically; costa with 2^nd^ (between R_1_ and R_2+3_), 3^rd^ (between R_2+3_ and R_4+5_), and 4^th^ (between R_4+5_ and M_1_) sections in proportion of 9.7: 2.7: 2; r-m before middle of discal cell; ultimate and penultimate sections of M_1_ in proportion of 3: 2.3; ultimate section of CuA_1_ ~ 1/5 of penultimate. Haltere pale brown.

**Figures 36–40.**
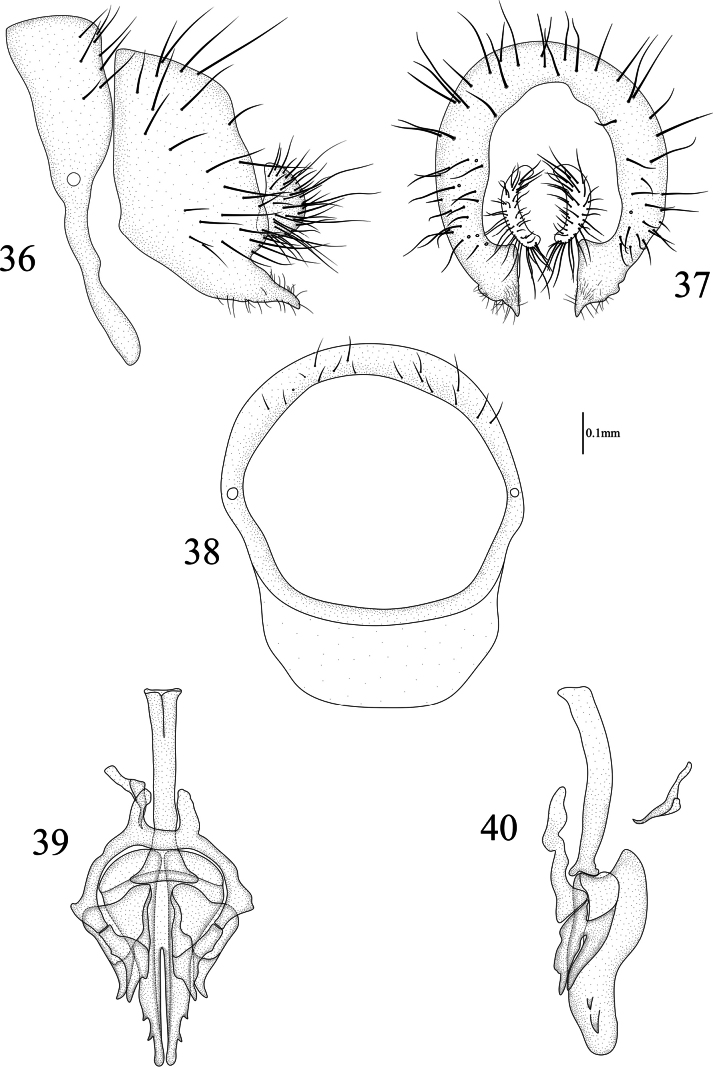
Homoneura (Homoneura) microtricha sp. nov. male **36** syntergosternite and epandrium, lateral view **37** epandrial complex, posterior view **38** syntergosternite, anterior view **39** phallic complex, ventral view **40** phallic complex, lateral view. Scale bar: 0.1 mm.

***Abdomen*** (Fig. 35) yellow, tergites 2–5 with pale brown posterior margin. Male genitalia (Figs 36–40): syntergosternite circular, with a trapeziform ventral process and with several dorsal setulae. Epandrium broad in lateral view; surstylus hairy, subuliform in lateral view. Hypandrium H-shaped. Pregonite knife-like in ventral view, connected with postgonite; postgonite long and spine-like in lateral view. Phallus with two pairs of subapical teeth in lateral view. Phallapodeme as long as phallus.

**Female.** Body length 5.9–6.3 mm, wing length 6.0–6.2 mm.

##### Distribution.

China (Chongqing).

##### Remarks.

The new species resembles Homoneura (Homoneura) longiacutata in the habitus and surstylus that is subuliform in lateral view [see Gao and Shi 2019: figs 35, 41–42], but it can be distinguished from the latter by the following: arista with longest ray as long as height of first flagellomere; ultimate section of CuA_1_ ~ 1/5 of penultimate; hypandrium H-shaped; postgonite long and spine-like in lateral view; phallus not curved backwards apically, without acute tip in lateral view. In H. (H.) longiacutata, arista with longest ray as long as 1/2 height of first flagellomere; ultimate section of CuA_1_ ~ 1/9 of penultimate; hypandrium inverse U-shaped; postgonite short, with two teeth-like processes in lateral view; phallus curved backwards apically and acute at apex in lateral view [see Gao and Shi 2019: figs 36, 40, 44, 45].

#### ﻿ Homoneura (Homoneura) multiseta

Taxon classificationAnimaliaDipteraLauxaniidae

Chen & Li sp. nov.

0D544508-1404-5E1A-9B27-053413A84C85

https://zoobank.org/8749E02C-8EC1-4C65-93E8-6478A74D82D4

Figs 41–45 , 46–50 Chinese name: 多鬃同脉缟蝇

##### Type material.

***Holotype***: ♂, **China**, Chongqing City, Jiangjin District, Simianshan Natural Reserve, Dahonghai, 28°35'34.27"N, 106°26'34.93"E, 1144 m, 15.VII.2022, leg. Xulong Chen. ***Paratypes***: 1♂, **China**, Chongqing City, Jiangjin District, Simianshan Natural Reserve, Zhengqiangou, 28°36'59.54"N, 106°26'25.88"E, 1273 m, 14.VI.2022, leg. Xulong Chen.

##### Etymology.

The specific name comes from the combination of the prefix *multi* and the Latin *seta*, referring to the epandrium covered by many setae.

##### Diagnosis.

Basal margin of brown apical spot on R_2+3_ at same vertical level as crossvein dm-cu; brown apical spots on R_2+3_, R_4+5_, and M_1_ slightly confluent. Male tergites 2–5 with brown posterior margin. Surstylus blunt and rolled up in ventral view. Hypandrium U-shaped. Pregonite broad and postgonite long, spine-like. Phallus with small sharp process in lateral view.

##### Description.

**Male.** Body length 8.7–8.8 mm, wing length 8.5 mm.

**Figures 41–45.**
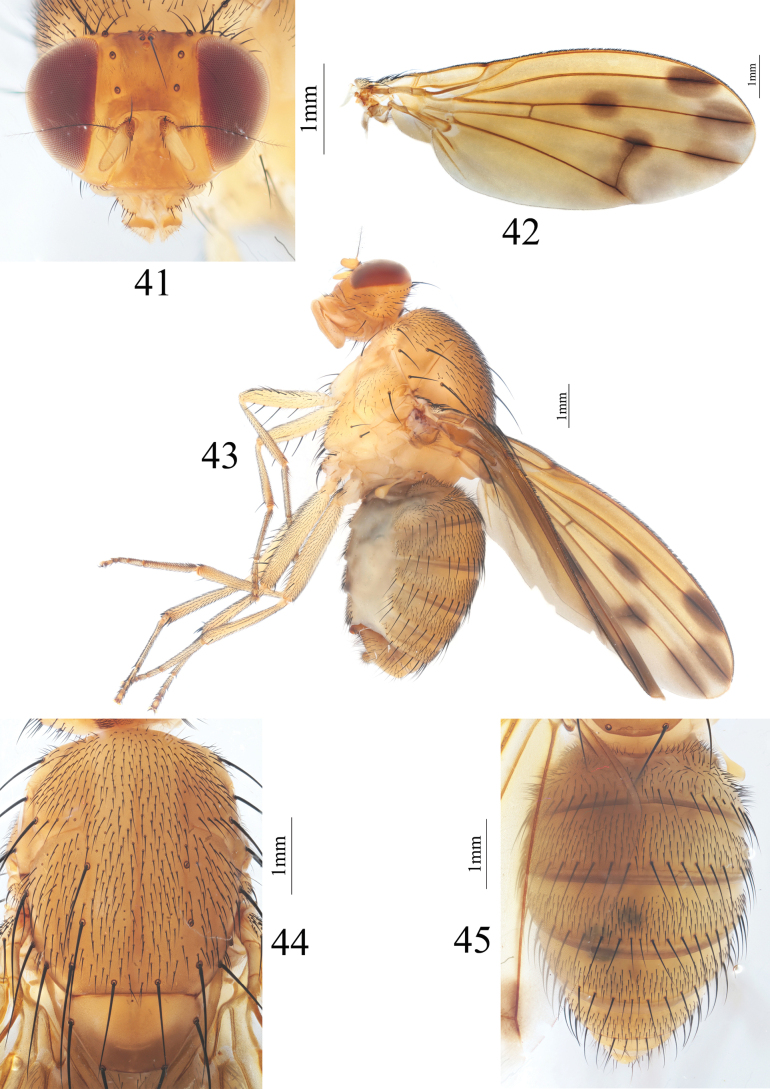
Homoneura (Homoneura) multiseta sp. nov. male **41** head, anterior view **42** wing **43** habitus, lateral view **44** thorax, dorsal view **45** abdomen, dorsal view.

***Head*** (Fig. 41) yellow. Frons as long as wide and parallel-sided; ocellar triangle yellow, ocellar seta developed. Gena ~ 1/8 height of eye. Antenna yellow, first flagellomere ~ 2.0 × longer than high; arista black except yellow at base, long plumose, with longest ray shorter than height of first flagellomere. Proboscis and palpus yellow.

***Thorax*** (Fig. 44) yellow, with gray pruinosity. 0+3 dorsocentral setae, anteriormost postsutural dorsocentral seta far from scutal suture, acrostichal setulae in ten rows. Legs yellow. Fore femur with nine posterior dorsal setae, four posterior ventral setae and ctenidium with 18 short setae; fore tibia with one dorsal preapical seta and one short apical ventral seta. Mid femur with five or six anterior setae and one apical posterior seta; mid tibia with one dorsal preapical seta and three strong apical ventral setae. Hind femur with several weak anterior ventral setae and one preapical anterior dorsal seta; hind tibia with one weak dorsal preapical seta and one short apical ventral seta. Wing (Fig. 42) slightly yellow, basal margin of brown apical spot on R_2+3_ at same vertical level as crossvein dm-cu; brown apical spots on R_2+3_, R_4+5_, and M_1_ slightly confluent and forming pale brown connecting area between apical spots on R_2+3_, R_4+5_, and M_1_; brown median spot on R_4+5_ separated from brown cloud-like spot on crossvein dm-cu; subcostal cell hyaline; seven short hairs present at base of R_4+5_; costa with 2^nd^ (between R_1_ and R_2+3_), 3^rd^ (between R_2+3_ and R_4+5_), and 4^th^ (between R_4+5_ and M_1_) sections in proportion of 4: 1: 0.8; r-m before middle of discal cell; ultimate and penultimate sections of M_1_ in proportion of 1: 1; ultimate section of CuA_1_ ~ 1/7 of penultimate. Haltere yellow.

**Figures 46–50.**
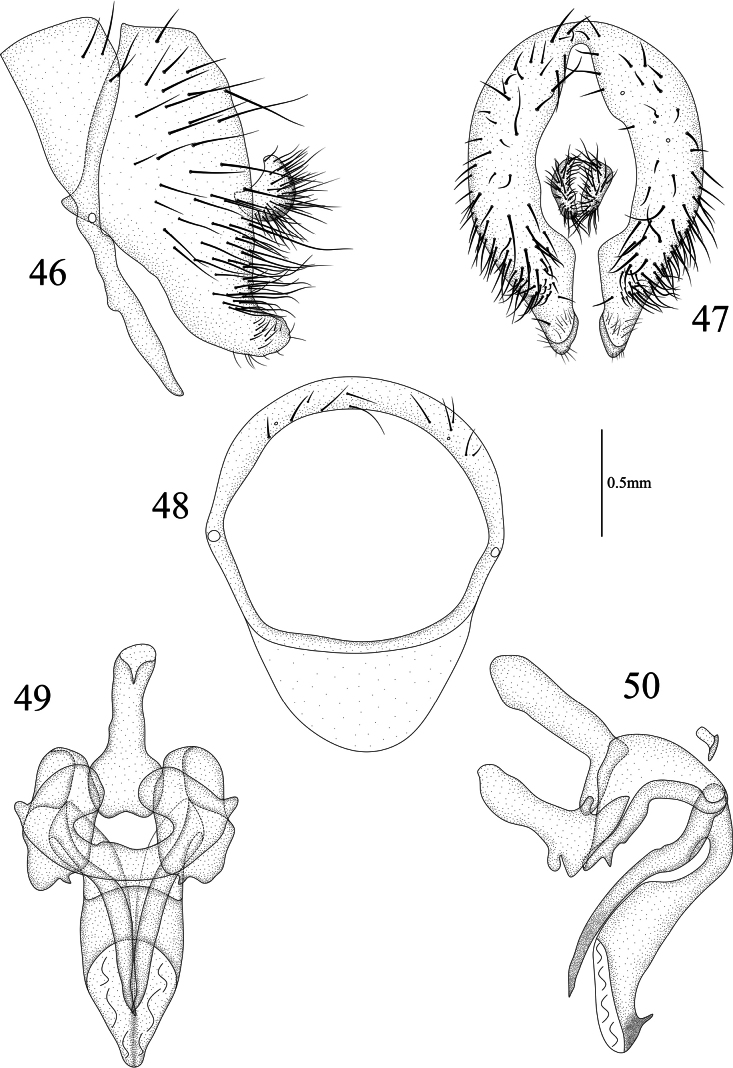
Homoneura (Homoneura) multiseta sp. nov. male **46** syntergosternite and epandrium, lateral view **47** epandrial complex, posterior view **48** syntergosternite, anterior view **49** phallic complex, ventral view **50** phallic complex, lateral view. Scale bar: 0.5 mm.

***Abdomen*** (Fig. 45) yellow, tergites 2–5 with brown posterior margin. Male genitalia (Figs 46–50): syntergosternite circular with a trapeziform ventral process, with several dorsal setulae. Epandrium broad in lateral view; surstylus blunt in lateral view, hairy and rolled up in ventral view. Hypandrium U-shaped. Pregonite broad, with a small tooth apically, postgonite long spine-like. Phallus curved backwards, with a small sharp process in lateral view. Phallapodeme shorter than phallus.

**Female.** Unknown.

##### Distribution.

China (Chongqing).

##### Remarks.

The new species resembles Homoneura (Homoneura) shunhuangshana in the habitus, basal margin of brown apical spot on R_2+3_ at same vertical level as crossvein dm-cu and mesonotum with acrostichal setulae in ten rows [see Chen and Li 2022: figs 7B–D], but it can be distinguished from the latter by the following: subcostal cell hyaline; brown apical spots on R_2+3_ shorter, as long as 1/2 length of ultimate section of M_1_; brown median spot on R_4+5_ behind middle point of distance between r-m and dm-cu; surstylus blunt, rolled up in ventral view; hypandrium U-shaped. In H. (H.) shunhuangshana, subcostal cell pale brown apically; brown apical spots on R_2+3_ longer, at least 2/3 length of ultimate section of M_1_; brown median spot on R_4+5_ at middle point of distance between r-m and dm-cu; surstylus horn-shaped in lateral view; hypandrium H-shaped [see Chen and Li 2022: figs 7B, 8A, D].

#### ﻿ Homoneura (Homoneura) serrulata

Taxon classificationAnimaliaDipteraLauxaniidae

Chen & Li sp. nov.

16C278CD-FA3B-5091-AADF-B7D046B5F13B

https://zoobank.org/0D01DD62-421D-43DC-8745-9EE3F678F714

Figs 51–55 , 56–60 Chinese name: 锯缘同脉缟蝇

##### Type material.

***Holotype***: ♂, **China**, Chongqing City, Jiangjin District, Dayuandong National Forest Park, Shuijingwan, 28°53'10.96"N, 106°14'19.32"E, 717 m, 13.VII.2022, leg. Xulong Chen. ***Paratypes***: 1♂, **China**, Chongqing City, Jiangjin District, Dayuandong National Forest Park, Tian’ehu, 28°52'54.45"N, 106°15'14.53"E, 728 m, 13.VII.2022, leg. Xulong Chen.

##### Etymology.

The specific name refers to the inner process of surstylus with serrulate margin in posterior view.

##### Diagnosis.

Basal margin of brown apical spot on R_2+3_ at same vertical level as crossvein dm-cu. Male tergites 2–5 with blackish brown posterior margin. Syntergosternite with a setula around spiracle. Inner process of surstylus evaginable apically with serrulate margin in posterior view, outer process long spine-like. Pregonite crossed at front of phallus in ventral view, postgonite digitiform and curved apically. Phallus tapering apically in lateral view.

##### Description.

**Male.** Body length 7.7 mm, wing length 7.8–7.9 mm.

**Figures 51–55.**
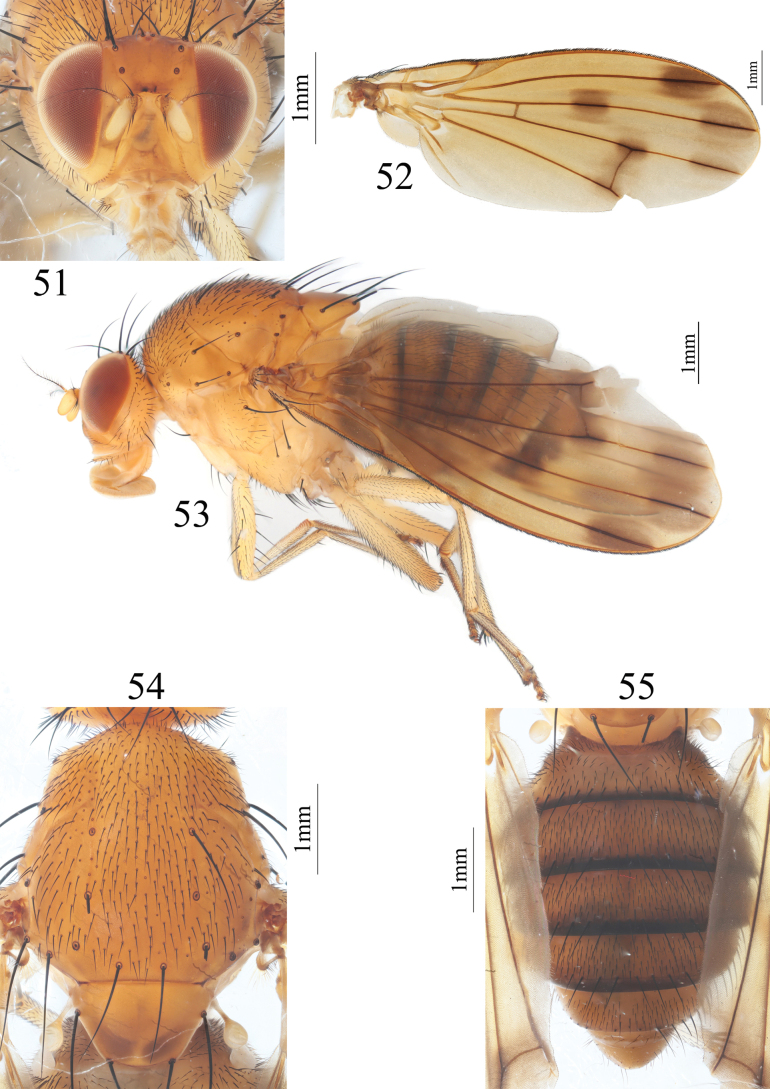
Homoneura (Homoneura) serrulata sp. nov. male **51** head, anterior view **52** wing **53** habitus, lateral view **54** thorax, dorsal view **55** abdomen, dorsal view.

Head (Fig. 51) yellow. Frons as long as wide and parallel-sided; ocellar triangle yellow, ocellar seta developed. Gena ~ 1/10 height of eye. Antenna yellow, first flagellomere ~ 2.0 × longer than high; arista black except yellow at base, long plumose, with longest ray as long as height of first flagellomere. Proboscis pale yellow, palpus yellow.

***Thorax*** (Fig. 54) yellow, with gray pruinosity. 0+3 dorsocentral setae, anteriormost postsutural dorsocentral seta far from scutal suture, acrostichal setulae in ten rows. Legs yellow. Fore femur with eight posterior dorsal setae, four or five posterior ventral setae and ctenidium with 14 short setae; fore tibia with one dorsal preapical seta and one short apical ventral seta. Mid femur with five or six anterior setae and one apical posterior seta; mid tibia with one dorsal preapical seta and three strong apical ventral setae. Hind femur with several weak anterior ventral setae and one preapical anterior dorsal seta; hind tibia with one weak dorsal preapical seta and one short apical ventral seta. Wing (Fig. 52) slightly yellow, basal margin of brown apical spot on R_2+3_ at same vertical level as crossvein dm-cu; brown apical spots on R_2+3_, R_4+5_, and M_1_ slightly confluent and forming pale brown connecting area between apical spots on R_2+3_, R_4+5_, and M_1_; brown median spot on R_4+5_ separated from brown cloud-like spot on crossvein dm-cu; subcostal cell pale brown apically; seven short hairs present at base of R_4+5_; costa with 2^nd^ (between R_1_ and R_2+3_), 3^rd^ (between R_2+3_ and R_4+5_), and 4^th^ (between R_4+5_ and M_1_) sections in proportion of 7.5: 2: 1.5; r-m before middle of discal cell; ultimate and penultimate sections of M_1_ in proportion of 1.1: 1.2. Haltere yellow.

**Figures 56–60.**
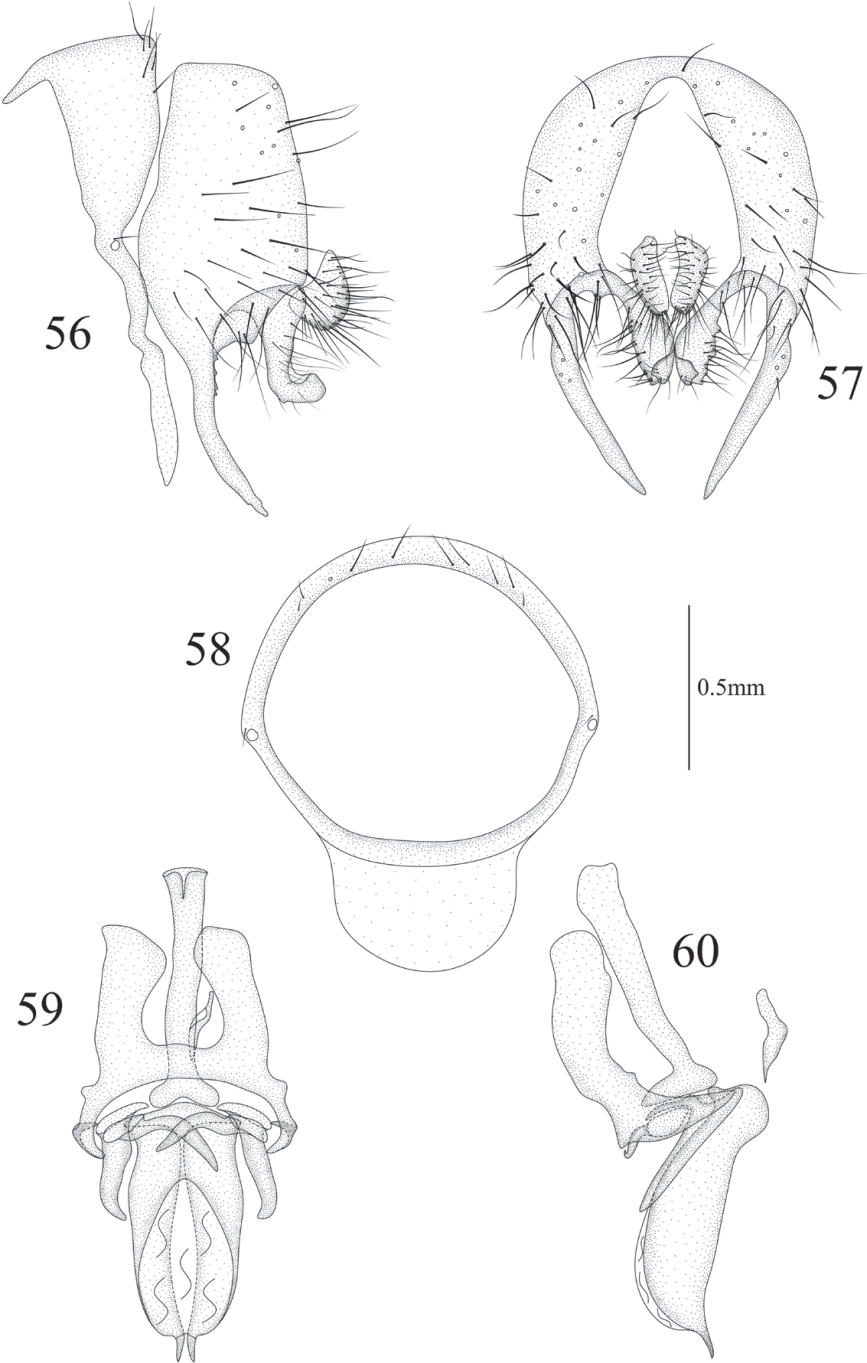
Homoneura (Homoneura) serrulata sp. nov. male **56** syntergosternite and epandrium, lateral view **57** epandrial complex, posterior view **58** syntergosternite, anterior view **59** phallic complex, ventral view **60** phallic complex, lateral view. Scale bar: 0.5 mm.

***Abdomen*** (Fig. 55) yellow, tergites 2–5 with blackish brown posterior margin. Male genitalia (Figs 56–60): syntergosternite circular with a trapeziform ventral process, with several dorsal setulae and setula around spiracle. Epandrium broad in lateral view; surstylus consisting of inner process and outer process, inner process evaginable apically with serrulate margin in posterior view, outer process long spine-like. Hypandrium H-shaped. Pregonite acute apically, crossed at front of phallus in ventral view, postgonite digitiform and curved apically. Phallus tapering apically in lateral view. Phallapodeme shorter than phallus.

**Female.** Unknown.

##### Distribution.

China (Chongqing).

##### Remarks.

The new species resembles Homoneura (Homoneura) anadaequata in the habitus, basal margin of brown apical spot on R_2+3_ at same vertical level as crossvein dm-cu and mesonotum with acrostichal setulae in ten rows [see Gao and Shi 2019: figs 1, 4, 7], but it can be distinguished from the latter by the following: brown apical spots on R_2+3_, R_4+5_, and M_1_ slightly confluent; syntergosternite with a setula around spiracle; inner process of surstylus evaginable apically with serrulate margin in posterior view; postgonites symmetrical in ventral view. In H. (H.) anadaequata, brown apical spots on R_4+5_ and M_1_ confluent, separated from apical spot on R_2+3_; syntergosternite without setula around spiracle; surstylus long and furcated in posterior view; postgonites asymmetrical in ventral view [see Gao and Shi 2019: figs 7, 9, 10, 11].

#### ﻿ Homoneura (Homoneura) simianshana

Taxon classificationAnimaliaDipteraLauxaniidae

Chen & Li sp. nov.

F086B010-96B6-515C-B2EE-4785712DDD12

https://zoobank.org/10AC3A27-80B5-41BD-8E58-221DAE4F0838

Figs 61–65 , 66–70 Chinese name: 四面山同脉缟蝇

##### Type material.

***Holotype***: ♂, **China**, Chongqing City, Jiangjin District, Simianshan Natural Reserve, Dawopu, 28°34'11.28"N, 106°20'26.96"E, 1007 m, 6.IX.2022, leg. Xulong Chen. ***Paratypes***: 3♂♂, same data as holotype; 4♂♂1♀, **China**, Chongqing City, Jiangjin District, Simianshan Natural Reserve, Zhengqiangou, 28°36'59.54"N, 106°26'25.88"E, 1273 m, 14.VI.2022, leg. Xulong Chen; 4♂♂3♀♀, **China**, Chongqing City, Jiangjin District, Simianshan Natural Reserve, Tudiyan, 28°37'23.62"N, 106°24'4.02"E, 1128 m, 7.IX.2022, leg. Xulong Chen; 1♂, **China**, Chongqing City, Jiangjin District, Simianshan Natural Reserve, Qinjiagou, 28°37'6.32"N, 106°23'53.40"E, 1131 m, 15.VII.2022, leg. Xulong Chen; 1♂, **China**, Chongqing City, Jiangjin District, Simianshan Natural Reserve, Zhenzhutan, 28°35'50.74"N, 106°25'25.70"E, 1226 m, 15.VII.2022, leg. Xulong Chen; 1♂, **China**, Chongqing City, Jiangjin District, Simianshan Natural Reserve, Dahonghai, 28°35'34.27"N, 106°26'34.93"E, 1144 m, 15.VII.2022, leg. Xulong Chen; 1♂1♀, **China**, Chongqing City, Jiangjin District, Dayuandong National Forest Park, Diaojiaolou, 28°52'16.19"N, 106°15'18.47"E, 759 m, 8.IX.2022, leg. Xulong Chen; 1♂, **China**, Chongqing City, Jiangjin District, Dayuandong National Forest Park, Diaojiaolou, 28°53'5.89"N, 106°15'42.18"E, 731 m, 13.VII.2022, leg. Xulong Chen.

##### Etymology.

The specific name refers to the type locality Simianshan Natural Reserve.

##### Diagnosis.

Basal margin of brown apical spot on R_2+3_ at same vertical level as crossvein dm-cu; brown apical spots on R_2+3_, R_4+5_, and M_1_ slightly confluent. Male tergites 2–5 with brown posterior margin. Surstylus inwardly curved apically in posterior view. Postgonite consisting of a pair of asymmetric sclerites, furcated in lateral view.

##### Description.

**Male.** Body length 8.6–8.8 mm, wing length 8.5–8.6 mm.

**Figures 61–65.**
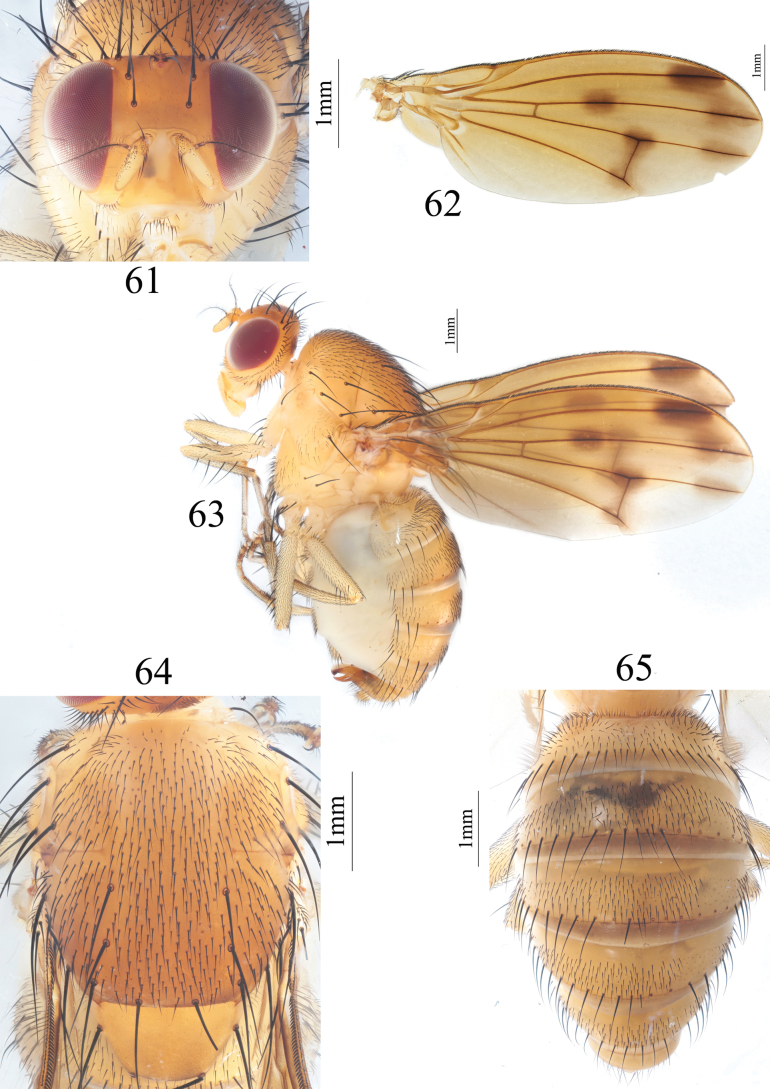
Homoneura (Homoneura) simianshana sp. nov. male **61** head, anterior view **62** wing **63** habitus, lateral view **64** thorax, dorsal view **65** abdomen, dorsal view.

***Head*** (Fig. 61) yellow. Frons as long as wide and parallel-sided; ocellar triangle yellow, ocellar seta developed, slightly longer than anterior fronto-orbital seta, anterior fronto-orbital seta shorter than posterior fronto-orbital seta. Gena ~ 1/6 height of eye. Antenna yellow, first flagellomere ~ 2.0 × longer than high; arista black except brown at base, long plumose, with longest ray slightly shorter height of first flagellomere. Proboscis pale yellow; palpus yellow.

***Thorax*** (Fig. 64) yellow, with gray pruinosity. 0+3 dorsocentral setae, anteriormost postsutural dorsocentral seta far from scutal suture, acrostichal setulae in ten irregular rows. Legs yellow. Fore femur with nine posterior dorsal setae, four posterior ventral setae. and ctenidium with 18–20 short setae; fore tibia with one dorsal preapical seta and one short apical ventral seta. Mid femur with five anterior setae and one apical posterior seta; mid tibia with one dorsal preapical seta and three strong apical ventral setae. Hind femur with several weak anterior ventral setae and one preapical anterior dorsal seta; hind tibia with one dorsal preapical seta and one short apical ventral seta. Wing (Fig. 62) slightly yellow, basal margin of brown apical spot on R_2+3_ at same vertical level as crossvein dm-cu; brown apical spots on R_2+3_, R_4+5_, and M_1_ slightly confluent and forming pale brown area between apical spots on R_2+3_, R_4+5_, and M_1_; brown median spot on R_4+5_ separated from brown cloud-like spot on crossvein dm-cu; subcostal cell pale brown apically; four short hairs present at base of R_4+5_, costa with 2^nd^ (between R_1_ and R_2+3_), 3^rd^ (between R_2+3_ and R_4+5_), and 4^th^ (between R_4+5_ and M_1_) sections in proportion of 5.5: 1.5: 1; r-m before middle of discal cell; ultimate and penultimate sections of M_1_ in proportion of 1: 1; ultimate section of CuA_1_ ~ 1/8 of penultimate. Haltere yellow.

**Figures 66–70.**
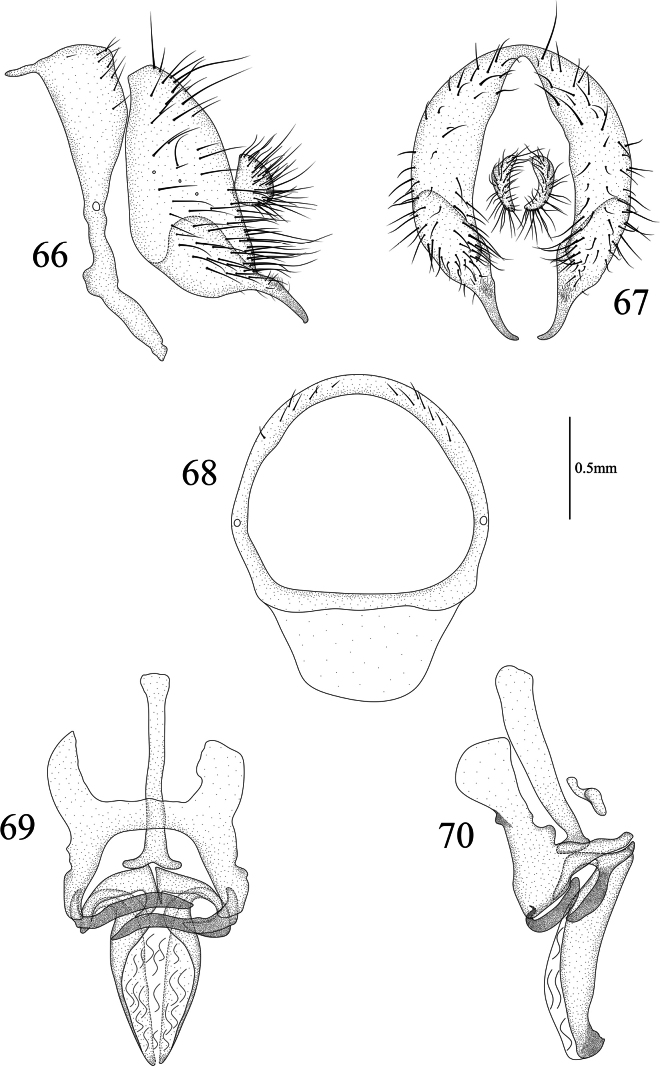
Homoneura (Homoneura) simianshana sp. nov. male **66** syntergosternite and epandrium, lateral view **67** epandrial complex, posterior view **68** syntergosternite, anterior view **69** phallic complex, ventral view **70** phallic complex, lateral view. Scale bar: 0.5 mm.

***Abdomen*** (Fig. 65) yellow, tergites 2–5 with brown posterior margin. Male genitalia (Figs 66–70): syntergosternite circular, with a trapeziform ventral process and with several dorsal setulae. Epandrium broad in lateral view; surstylus long digitiform in lateral view, inwardly curved apically in posterior view. Hypandrium H-shaped. Pregonite long digitiform and curved apically in ventral view, postgonite consisting of a pair of asymmetric sclerites, furcated in lateral view. Phallus curved backwards in lateral view. Phallapodeme as long as phallus.

**Female.** Body length 8.8 mm, wing length 8.5–8.6 mm.

##### Distribution.

China (Chongqing).

##### Remarks.

The new species resembles Homoneura (Homoneura) shunhuangshana in the habitus, mesonotum with acrostichal setulae in ten rows, basal margin of brown apical spot on R_2+3_ at same vertical level as crossvein dm-cu and tergites 2–5 each with brown posterior margin [see Chen and Li 2022: figs 7B–E], but it can be distinguished from the latter by the following: fore femur with nine posterior dorsal setae, four posterior ventral setae; mid femur with five anterior setae; brown apical spots on R_2+3_ shorter, as long as 1/2 length of ultimate section of M_1_; brown median spot on R_4+5_ behind middle point of distance between r-m and dm-cu. In H. (H.) anadaequata, fore femur with eight posterior dorsal setae, six posterior ventral setae; mid femur with six or seven anterior setae; brown apical spots on R_2+3_ longer, at least 2/3 length of ultimate section of M_1_; brown median spot on R_4+5_ at middle point of distance between r-m and dm-cu [see Chen and Li 2022: fig. 7B, C].

## ﻿Discussion

Homoneura (Homoneura) henanensis species group is species rich in Oriental and Palearctic species, with 53 described species, and is now the largest group of the subgenus Homoneura. The species of the *henanensis* group are almost unified in external morphological characters (i.e., large body size, antennal first flagellomere ~ 2.0 × longer than high, wing with five large brown spots), but are not sufficient for species identification. Interestingly, the male genitalia of *henanensis* group are extremely complex and variable, and different species exhibit distinctive shapes and forms; therefore, the male genitalia structures can provide the most reliable diagnostic characters for species delimitation, while the value of the female genitalia is relatively limited for species identification. Seven new species of the *henanensis* group are described in this paper, of which six species have extremely characteristic male genitalia, except for H. (H.) microtricha sp. nov. that resembles H. (H.) longiacutata in the shape of surstylus, but the new species can be separated from the latter by the following male genitalia characters: 1) the H-shaped hypandrium; 2) the long spine-like postgonite in lateral view; 3) the not curved phallus, blunt and round apically. In H. (H.) longiacutata, the hypandrium is U-shaped, the postgonite is short and the middle part depressed, and the phallus is curved backwards apically and acute at the apex.

**Figure 71.**
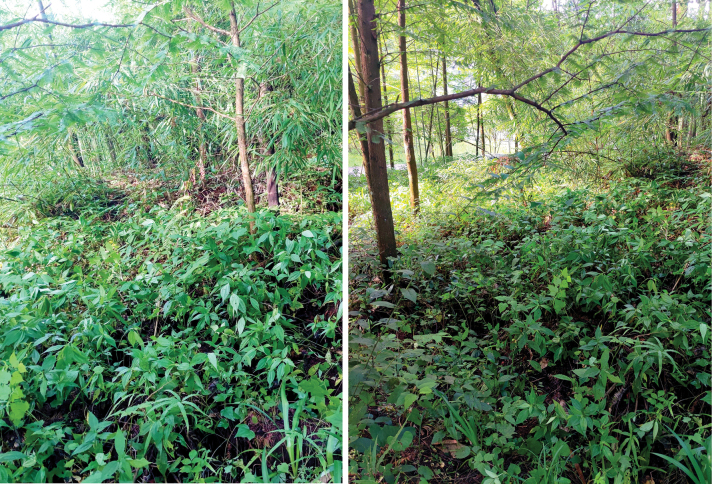
Habitat and plants in Simianshan Natural Reserve, Zhengqiangou.

The *henanensis* group is diverse in Chinese moist and shaded herb habitats (Fig. 71; including Shaanxi, Chongqing, Henan, Guangdong, Guangxi, Zhejiang), and suitable habitats can attract a large number of species that survive and reproduce here, which may be the reason they are abundant in collections, as well as contribute to the discovery of new species. However, it is surprising that only one species of this group has been found in Yunnan province, which has the highest species richness of lauxaniid flies in China; such distributional gaps may be the result of insufficient sampling for this group. There is no doubt that additional species await discovery in unsampled primary forests throughout southwestern China.

## Supplementary Material

XML Treatment for Homoneura (Homoneura) biconica

XML Treatment for Homoneura (Homoneura) dilatata

XML Treatment for Homoneura (Homoneura) jiangjinensis

XML Treatment for Homoneura (Homoneura) microtricha

XML Treatment for Homoneura (Homoneura) multiseta

XML Treatment for Homoneura (Homoneura) serrulata

XML Treatment for Homoneura (Homoneura) simianshana

## ﻿Additional information

### Author ORCIDs

Xulong Chen https://orcid.org/0000-0003-0787-3333

Pengyan You https://orcid.org/0000-0002-7485-8056

Wenliang Li https://orcid.org/0000-0001-9019-1223

Zhisheng Zhang https://orcid.org/0000-0002-9304-1789

### Data availability

All of the data that support the findings of this study are available in the main text.

## References

[B1] Álvarez-Padilla F Hormiga G (2007) A protocol for digesting internal soft tissues and mounting spiders for scanning electron microscopy. The Journal of Arachnology 35(3): 538–542. 10.1636/Sh06-55.1

[B2] Chen XL Li WL (2022) Four new species of the subgenus Homoneura from Hunan Province, China (Diptera: Lauxaniidae: *Homoneura*). Oriental Insects 56(4): 437–462. 10.1080/00305316.2021.2022546

[B3] Cumming JM Wood DM (2017) 3. Adult morphology and terminology. In: Kirk-Spriggs AH Sinclair BJ (Eds) Manual of Afrotropical Diptera: Vol.1. Suricata 4. South African National Biodiversity Institute, Pretoria, 89–133.

[B4] Gaimari SD Miller RM (2021) 74. Lauxaniidae (Lauxaniid flies). In: Kirk-Spriggs AH Sinclair BJ (Eds) Manual of Afrotropical Diptera.Vol. 3. Brachycera—Cyclorrhapha, excluding Calyptratae. Suricata 8. South African National Biodiversity Institute, Pretoria, 1757–1781.

[B5] Gao XF Shi L (2019) Nine new species of genus *Homoneura* from Qinling mountains in China (Diptera: Lauxaniidae). Zootaxa 4608(3): 401–432. 10.11646/zootaxa.4608.3.1 31717130

[B6] Gao CX Yang D (2002) A review of the genus *Homoneura* from Guizhou, China (Diptera: Lauxaniidae). Annales Zoologici 52(2): 293–296.

[B7] Gao CX Yang D (2003) A revision of the genus *Homoneura* from Tibet, China (Diptera, Lauxaniidae). Deutsche Entomologische Zeitschrift 50(2): 243–248. 10.1002/mmnd.20030500208

[B8] Gao CX Yang D (2004) A review of the genus *Homoneura* from Guangxi, China (Diptera: Lauxaniidae). The Raffles Bulletin of Zoology 52(2): 351–364.

[B9] Gao CX Yang D (2005) Notes on genus *Homoneura* from Guizhou, China (Diptera: Lauxaniidae). Zootaxa 1010(1): 15–24. 10.11646/zootaxa.1010.1.2

[B10] Kertész K (1915) H. Sauter’s Formosa Ausbeute. Lauxaniidae (Dipt). II. Annales Musei National Hungarici 13: 491–534.

[B11] Li WL Yang D (2012) Eleven new species of the Homoneura (Homoneura) beckeri group from Yunnan, China (Diptera, Lauxaniidae). Zootaxa 3537(1): 1–28. 10.11646/zootaxa.3537.1.1

[B12] Li WL Yang D (2013) Four new species of *Homoneura* s. str. from Yunnan, China (Diptera, Lauxaniidae). Revue Suisse de Zoologie 120(4): 549–561.

[B13] Li WL Yang D (2015) Species of the subgenus Neohomoneura Malloch from Yunnan, China (Diptera, Lauxaniidae). Transactions of the American Entomological Society 141(1): 27–44. 10.3157/061.141.0104

[B14] Li WL Qi L Yang D (2019) First record of the genus *Trypetisoma* Malloch, 1924 (Diptera, Lauxaniidae) for China with nine species. Zootaxa 4608(1): 035–064. 10.11646/zootaxa.4608.1.2 31717159

[B15] Li WL Chen XL Yang D (2020a) Five new species of the genus *Noeetomima* Enderlein (Diptera: Lauxaniidae) from China, with a key to world species. Zootaxa 4768(4): 499–516. 10.11646/zootaxa.4768.4.3 33055636

[B16] Li WL Chen XL Yang D (2020b) Four new species of the subgenus Minettiella from China (Diptera, Lauxaniidae, *Minettia*). ZooKeys 932: 93–111. 10.3897/zookeys.932.50763 32476975 PMC7237505

[B17] Li WL Qi L Yang D (2020c) Four new species of the subfamily Homoneurinae (Diptera, Lauxaniidae) from southwestern China. ZooKeys 953: 119–136. 10.3897/zookeys.953.53976 32821199 PMC7398949

[B18] Li WL Qi L Yang D (2020d) First Chinese record and the second species of the genus *Pleurigona* Malloch, 1929 (Diptera: Lauxaniidae). Revue Suisse de Zoologie 127(2): 245–248. 10.35929/RSZ.0018

[B19] Li WL Qi L Yang D (2020e) Four species of the genus *Lauxania* Latreille, 1804 (Diptera: Lauxaniidae) from China. Oriental Insects 54(3): 417–432. 10.1080/00305316.2019.1665595

[B20] Li WL Qi L Yang D (2020f) Four new species of the subgenus Frendelia Collin, 1948 (Diptera: Lauxaniidae: *Minettia*) from China. Annales Zoologici 70(2): 273–284. 10.3161/00034541ANZ2020.70.2.007

[B21] Li WL Chen XL Feng KL Zhao SJ Yang D (2021a) Four new species of the genus *Luzonomyza* Malloch (Diptera, Lauxaniidae) from China. ZooKeys 1074: 43–59. 10.3897/zookeys.1074.68392 34963752 PMC8654813

[B22] Li WL Qi L Yang D (2021b) Three new species of the genus *Noonamyia* Stuckenberg 1971 (Diptera: Lauxaniidae) from China. Oriental Insects 55(1): 119–132. 10.1080/00305316.2020.1754955

[B23] Li WL Bai YM Yang D (2023) The genus *Minettia* Robineau-Desvoidy, 1830 (Diptera: Lauxaniidae: Lauxaniinae) from the Oriental Region with descriptions of seventeen new species. Zootaxa 5256(3): 201–249. 10.11646/zootaxa.5256.3.1 37045228

[B24] Malloch JR (1926) A new species of Sapromyzidae from China (Diptera). Bulletin of the Brooklyn Entomological Society 21: 176.

[B25] Malloch JR (1927) Fauna sumatrensis. Sapromyzidae (Diptera). Supplementa Entomologica 15: 102–110.

[B26] Matsumura S (1916) Thousand Insects of Japan. Additamenta. 2 (Diptera): 185–474.

[B27] Papp L Gaimari SD (2013) The holotype of *Homoneuragrandis* (Kertész, 1915) with description of a new species from Taiwan (Diptera, Lauxaniidae). Acta Zoologica Academiae Scientiarum Hungaricae 59(1): 31–39.

[B28] Shi L Yang D (2014) Supplements to species groups of the subgenus Homoneura in China (Diptera: Lauxaniidae: *Homoneura*), with descriptions of twenty new species. Zootaxa 3890(1): 1–117. 10.11646/zootaxa.3890.1.1 25544372

[B29] Shi L Yang D (2015) Two new species of the genus *Luzonomyza* from China (Diptera, Lauxaniidae). Zootaxa 3964(1): 087–094. 10.11646/zootaxa.3964.1.5 26249422

[B30] Shi L Li WL Yang D (2009) Five new species of the genus *Dioides* from China (Diptera: Lauxaniidae). Annales Zoologici 59(1): 93–105. 10.3161/000345409X432600

[B31] Shi L Gaimari SD Yang D (2017a) Five new species of the genus *Tetroxyrhina* Hendel from China (Diptera, Lauxaniidae). Zootaxa 4247(3): 246–280. 10.11646/zootaxa.4247.3.2 28610070

[B32] Shi L Gao XF Shen RR (2017b) Four new species of the subgenus Homoneura from Jiangxi Province, China (Diptera: Lauxaniidae: *Homoneura*). Zootaxa 4365(3): 361–377. 10.11646/zootaxa.4365.3.5 29686209

[B33] Shi L Liu M Hu ZK (2020) A new species of the genus *Noeetomima* Enderlein (Diptera, Lauxaniidae) from Guizhou, China with a key to worldwide species. ZooKeys 1000: 107–123. 10.3897/zookeys.1000.57577 33354137 PMC7736083

[B34] Wulp FM van der (1891) Eenige uitlandsche Diptera. Tijdschrift voor Entomologie 34: 193–218. [1890–1891]

[B35] Wang JC Gao CX Yang D (2012) Three new species of the Homoneura (Homoneura) henanensis species group (Diptera, Lauxaniidae), with a key to Chinese species. Zootaxa 3262(1): 35–45. 10.11646/zootaxa.3262.1.3

[B36] Yang D Zhu F Hu XY (1999) New species of Lauxaniidae from Henan (Diptera: Acalyptratae). 211–217. In: Shen XC Shi ZY (Eds) Fauna and Taxonomy of Insects in Henan: Vol.4: 211–217. China Agricultural Scientech Press, Beijing, 404 pp.

[B37] Yang D Hu XY Zhu F (2001) Diptera: Lauxaniidae. 446–453. In: Wu H, Pan CW (Eds) Insects of Tianmushan National Nature Reserve: 446–453. Science Press, Beijing, 764 pp.

[B38] Yang D Zhu F Hu XY (2003) Diptera: Lauxaniidae. 555–558. In: Huang BK (Ed.) Fauna of Insects in Fujian Province of China. 8. Fujian Science and Technology Publishing House, Fuzhou, 706 pp.

[B39] You PY Chen XL Li WL (2023) Four new species of the subgenus Homoneura from Yintiaoling Nature Reserve, China (Diptera: Lauxaniidae: *Homoneura*). Zootaxa 5257(1): 143–159. 10.11646/zootaxa.5257.1.11 37044615

[B40] Zhao SJ Chen XL Li WL Yang D (2022) Two new species of the genus *Melanopachycerina* (Diptera: Lauxaniidae) from China, with a key to known species worldwide. Annales Zoologici 72(4): 943–950. 10.3161/00034541ANZ2022.72.4.010

